# Defining how multiple lipid species interact with inward rectifier potassium (Kir2) channels

**DOI:** 10.1073/pnas.1918387117

**Published:** 2020-03-25

**Authors:** Anna L. Duncan, Robin A. Corey, Mark S. P. Sansom

**Affiliations:** ^a^Department of Biochemistry, University of Oxford, Oxford OX1 3QU, United Kingdom

**Keywords:** Kir channel, lipids, molecular dynamics, PIP2, PS

## Abstract

Protein–lipid interactions are a key element of the function of many integral membrane proteins. These potential interactions should be considered alongside the complexity and diversity of membrane lipid composition. Inward rectifier potassium channel (Kir) Kir2.2 has multiple interactions with plasma membrane lipids: Phosphatidylinositol (4, 5)-bisphosphate (PIP_2_) activates the channel; a secondary anionic lipid site has been identified, which augments the activation by PIP_2_; and cholesterol inhibits the channel. Molecular dynamics simulations are used to characterize in molecular detail the protein–lipid interactions of Kir2.2 in a model of the complex plasma membrane. Kir2.2 has been simulated with multiple, functionally important lipid species. From our simulations we show that PIP_2_ interacts most tightly at the crystallographic interaction sites, outcompeting other lipid species at this site. Phosphatidylserine (PS) interacts at the previously identified secondary anionic lipid interaction site, in a PIP_2_ concentration-dependent manner. There is interplay between these anionic lipids: PS interactions are diminished when PIP_2_ is not present in the membrane, underlining the need to consider multiple lipid species when investigating protein–lipid interactions.

## Significance

Ion channels form pores that allow for the selective transport of ions across cell membranes, generating electrical signals in response to a variety of signals. Inward rectifier potassium (Kir) channels in particular are regulated by direct interactions with the complex mixture of lipids that are present in eukaryotic cell membranes. However, the molecular details of these concurrent lipid interactions with Kir channels are not clear and difficult to access via experimental methods. Here, we simulate the Kir2.2 channel in a complex lipid mixture to explore how anionic phospholipids and cholesterol dynamically organize around the membrane protein. In particular we demonstrate a synergy between binding interactions of different anionic phospholipid species which are known to activate Kir channels.

Interactions of phospholipids and cholesterol (Chol) with ion channels, receptors, and other membrane proteins play a key role in the structure and function of these proteins (1). Structural, biophysical, and computational techniques have identified specific lipids undergoing tight interactions, which in many cases have been shown to have functional relevance (2–5). In particular, advances in cryoelectron microscopy including the use of nanodiscs are revealing an increasing number of functionally relevant binding sites for lipids (see e.g., ref. 6). There is also an increasing understanding of the complexity of lipid membranes, the diversity of such complexity, and the impact this lipid complexity has on membrane function, particularly on protein–lipid interactions (7–9).

Molecular dynamics (MD) simulations provide a powerful tool to investigate protein–lipid interactions. In particular, the coarse-grained MARTINI force field (10–13) has been widely applied, since it offers ready access to timescales on the order of tens of microseconds, which are particularly relevant for the study of protein–lipid interactions. The coarse-grained MD simulation approach has been used study protein–lipid interactions with a wide range of membrane proteins (14, 15). The agreement of computationally identified protein–lipid interactions sites with experimental data has demonstrated that, despite the loss of accuracy in coarse-grained models, the approach can successfully identify specific protein–lipid interactions sites. In particular, there has been good agreement with data from X-ray crystal structures [for, e.g., inward rectifier potassium (Kir) channels (16, 17), the ANT transporter protein (18, 19), and components of the mitochondrial redox chain (20, 21)], from cryoelectron microscopy structures (22), and from mass spectrometry (3, 23).

Analysis of protein–lipid interactions in MD simulations has been performed in several ways: by identifying protein residues that have high frequency of lipid contacts (e.g., ref. 24), by identification of interaction fingerprints (4), and by clustering residues that simultaneously interact frequently with lipid headgroup particles, binding sites can be identified (19–21). Recently, a methodology has been developed to use graph-theoretic community analysis (25) of simultaneously interacting residues, rather than the more straightforward cluster analysis referenced above. This community analysis approach was applied to examine Chol interactions with the Kir2.2 channel (26), which have previously proved difficult to definitively identify (27, 28).

Kir channels are regulated by several lipid species, including phosphatidylinositol 4,5-bisphosphate (PIP_2_), which activates mammalian Kir channels (29–31) [but inhibits the homologous bacterial KirBac channels (32)]. The crystal structure of Kir2.2 with PIP_2_ bound (33) suggests that activation occurs by the PIP_2_ headgroup interacting with both the transmembrane domain and the cytoplasmic domain, bringing them closer together and favoring a channel open conformation. There is also evidence for a secondary anionic lipid site (34). Electrophysiology studies show that there is enhanced activity of Kir2.1 and Kir2.2 when anionic lipids are present. Initial mutagenesis work implicated Kir2.1 residues K219 (equivalent of K220 in Kir2.2 channels) (34). Further work showed that the effect of secondary anionic lipid is ablated when residue K62 is mutated and restored when either K62 is mutated to tryptophan or when K62C is tethered to membrane, even without presence of anionic lipids (35, 36). It was hypothesized that the anionic lipid phosphatidylserine (PS) interacts with the so-called slide helix to bring this part of the structure adjacent to the membrane, and that this interaction can occur independently of the PIP_2_ interaction at the primary site (*SI Appendix*, Fig. S1). Together, the two types of lipid interaction enhance the open probability of the channel. The other major regulatory lipid is Chol, which inhibits Kir2.2 via direct interactions (37–39). A number of combined computational and experimental papers have identified Chol interaction sites (27, 28, 40–43), with the most recent study (26) having been discussed above. Interplay between the various lipid species that are present in the plasma membrane (PM) in regulation of Kir channels remains to be fully explored. It appears the Chol interaction sites do not overlap with those of PIP_2_ (44). However, few simulations have been performed with multiple key lipid species simultaneously present in the membrane environment (45).

Here we perform a set of large time- and length-scale simulations of Kir2.2 (20 to 50 µs for each simulation; total simulation time of >400 µs, with length scales of 30 to 130 nm, containing 9 or 144 proteins) in complex, asymmetric lipid bilayers containing PIP_2_, PS, and Chol, alongside other principal lipids of the mammalian PM, namely glycolipids, ceramides, phosphatidylethanolamine (PE), and phosphatidylcholine (PC). We identify multiple lipid interaction sites for each lipid type and extract kinetic information for each site. This analysis reveals the dynamic interplay of the two anionic lipids (PIP_2_ and PS) at their two binding sites (the primary and secondary anionic lipid sites).

Here, Kir2.2 channel–lipid interactions were determined from simulations in a bilayer containing multiple, functional lipid species. By applying analytical techniques to identify lipid interaction sites, introduced recently by Barbera et al. (26) to look at Chol interaction sites of Kir2.2, we can identify multiple lipid interaction sites a priori. Further, by calculating the residence times of each interaction site, we identify their kinetic profiles, while free energy perturbation calculations (see, e.g., ref. 46) quantify the preference for PIP_2_ vs. PS binding at each site. These analyses allow us to build up a picture of diverse lipid interactions on the surface of Kir2.2 channels, corresponding to the multimodal regulation of this channel by different lipid species.

## Results

Coarse-grained MD simulations of 20 to 40 µs were run with 144 copies of the Kir2.2 channel embedded in membranes with varied lipid compositions (*SI Appendix*, Table S1), including from a model of the PM (with an inner leaflet containing PC:PE:Chol:PS:PIP_2_ and an outer leaflet containing PC:PE:Chol:Sph:GM3), a PM with PIP_2_ removed, and a bilayer containing solely PC. In order to obtain a more complete understanding of the effect of varying lipid composition and of the possible interplay between lipids, additional (50 µs) simulations were run with nine copies of the Kir2.2 channel embedded in membranes with and without PIP_2_, with and without PS, with and without Chol, and with and without GM3 (*SI Appendix*, Table S1).

In order to understand which lipids cluster preferentially around Kir2.2, at which residues, and the kinetics of the interaction we performed calculations of the distribution of lipids around Kir2.2 channels, and of residue–lipid interaction frequencies. We subsequently identified lipid interaction sites on the Kir2.2 channel using network analysis and determined the residence times and number of simultaneous contacts between protein and lipid headgroups at each interaction site. To further characterize the anionic lipid preferences at the sites corresponding to experimentally determined primary and secondary interaction sites, we performed free energy perturbation (FEP) calculations.

### Anionic Lipids Are the Principal Interacting Species in the Inner Leaflet.

**Fig. 1.**
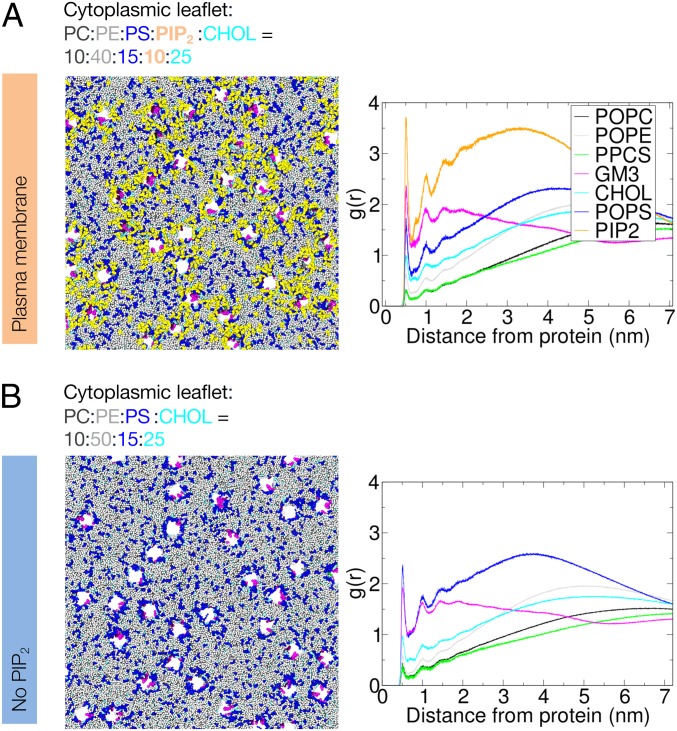
Simulations and lipid distributions. *A* shows data from the *Large System, PM* simulation (see *SI Appendix*, Table S1 for details), *B* shows data from the *Large System, No PIP*_*2*_ simulation. In each case the left shows a snapshot at 20 µs of the inner (cytoplasmic) leaflet face of the membrane, showing all lipids but with Kir2.2 channels removed, and the right shows the radial distribution of lipid species about Kir2.2 channels. From *A* it is evident that in the *PM* simulation PIP_2_ (yellow) clusters around the protein, both close to the TM region and in the area covered by the cytoplasmic domain. In contrast, for the *No PIP*_*2*_ simulation in *B*, PS (blue) can be seen to be clustering around the TM region of Kir2.2 channels.

Clustering of PIP_2_ and PS can be observed in simulations of Kir2.2 in the PM, with PS appearing to replace PIP_2_ if PIP_2_ is not included in the membrane (Fig. 1). In order to quantify the lipid clustering around Kir2.2 channels, radial distributions of each lipid species around all Kir2.2 channels were calculated (Fig. 1*A*). In the inner leaflet, PIP_2_ and PS are the principal lipid species in the first annular shell around Kir2.2, with PIP_2_ being the dominant species overall (Fig. 1*A* and *SI Appendix*, Fig. S2). In simulations where PIP_2_ is not included (Fig. 1*B* and *SI Appendix*, Fig. S2), PS is the principal lipid species of the inner leaflet that is enriched around Kir2.2 channels. These trends are apparent whether or not Chol is present (*SI Appendix*, Fig. S2). In the outer (i.e., extracellular) leaflet, GM3 is the principal lipid species interacting with Kir2.2, with a degree of first annular shell enrichment comparable to that of PIP_2_ and PS in the inner leaflet. This is of possible biological relevance given suggestions of the role of sphingolipids (which include gangliosides such as GM3) in the formation of outer leaflet nanodomains with possible roles in brain development and/or function (47). However, there is no appreciable enrichment of Chol (which might be expected to be colocalized with sphingolipids in nanodomains) around Kir2.2 channels, relative to bulk concentration, regardless of whether or not PIP_2_ or PS is present (*SI Appendix*, Fig. S2). PC, PE, and sphingomyelin are excluded from the first annular shell around Kir2.2.

### Interaction Hotspots Mapped for All Lipid Species.

**Fig. 2.**
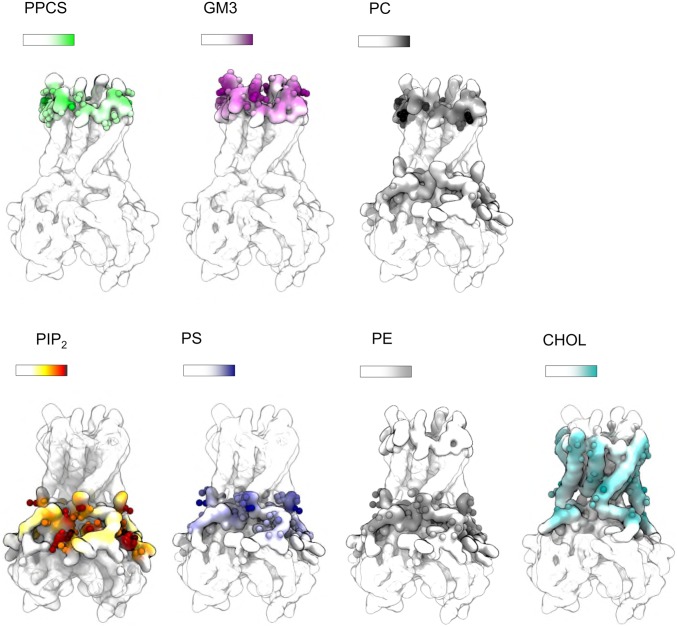
Lipid headgroup interactions in the PM simulation. The frequency of lipid headgroup interactions at each protein residue for all lipids present in the *Large System, PM* simulations (containing 144 Kir2.2 channels; see *SI Appendix*, Table S1 for details). Interactions of lipid headgroups are mapped onto the protein surface with the frequency of interaction at each residue colored on a sliding scale (transparent = no interaction; white = lowest interaction frequency; colored = high interaction frequency). Similar analysis for *No PIP*_*2*_ and *PC* simulations is shown in *SI Appendix*, Fig. S3. Residues that interact with a frequency >2.5% of the total lipid headgroup interactions (for a given lipid type) are show with side-chain beads as spheres. The definition of lipid headgroups is detailed in *Methods*.

In order to understand how specific lipid molecules interact with Kir2.2 channels, the frequency of lipid headgroup interactions at each residue of Kir2.2 was calculated and mapped onto the protein surface, thus revealing lipid headgroup interaction “hotspots” (Fig. 2 and *SI Appendix*, Figs. S3 and S4). PIP_2_ and PS headgroups interact with both the transmembrane domain, at the classical PIP_2_ interaction site, and with the cytoplasmic domain. Chol headgroup interactions occurred at residues closer to the membrane center and were more dispersed (the percentage of total Chol contacts for any individual residue was <4% of that for all residues, which is lower than that of PIP_2_, PS, or GM3 contacts; *SI Appendix*, Fig. S5). Chol headgroup interactions occurred in particular with residues P63, I67, R80, L83, L84, S87, I100, L103, I107, C155, P156, L157, F160, V164, and V168 (*SI Appendix*, Fig. S5), in agreement with those previously identified (26). In the outer leaflet, sphingomyelin and PC headgroups interact with similar residues on the protein surface. GM3 shows nonspecific interactions around Kir2.2 channels, but the extended glycan headgroup interacts further away from the bilayer center than do PC and sphingomyelin headgroups, close to the extracellular mouth of the channel. The GM3 headgroup interacts in particular with H108, E112, T119, F120, K121, and E154, with each of these residues accounting for over 6% of total GM3 headgroup contacts with the channel. To the best of our knowledge there are no glycosylation sites on Kir2.2 channels (see, e.g., https://www.rcsb.org/pdb/explore/remediatedSequence.do?structureId=3SPI). The interaction of the anionic ganglioside with cationic side chains close to the extracellular mouth, in particular at residues H108 and K121, might be anticipated to modulate the conductance of the channel.

Simulations of Kir2.2 embedded in a membrane containing only PC show that PC headgroups can also interact at some residues of the PIP_2_ interaction site when no anionic lipids are present (also see *SI Appendix*, Fig. S6). Similar behavior has been seen for the TRP channel PC2 (22).

### Anionic Lipids Interact, but Not Only at the Expected Primary and Secondary Sites.

**Fig. 3.**
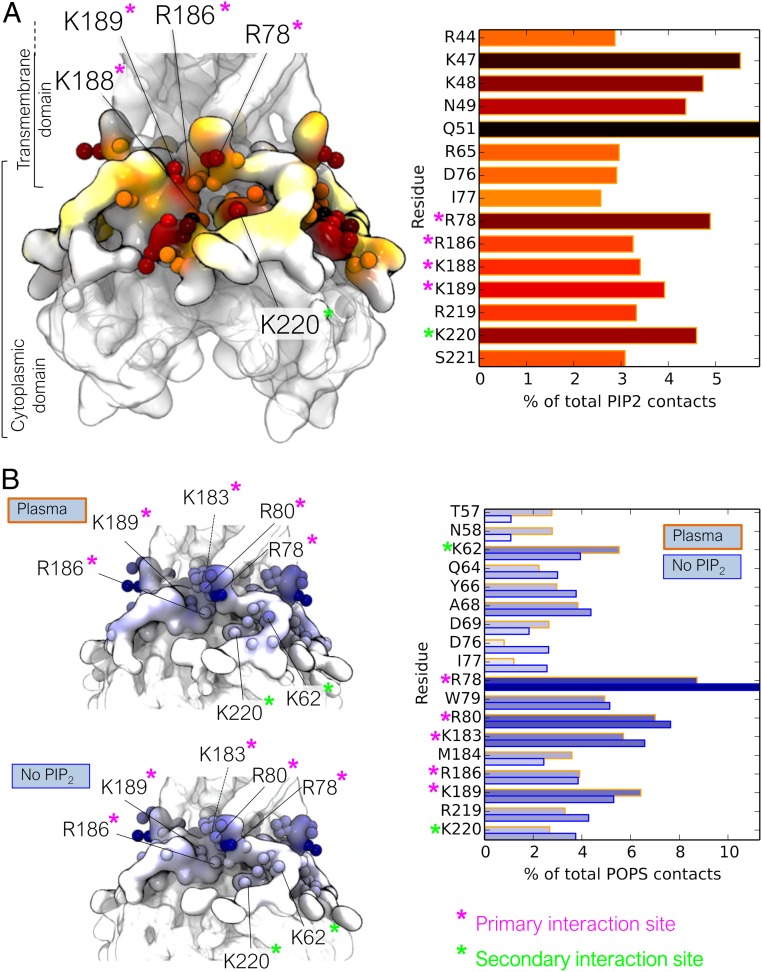
Frequency of PIP_2_ and PS headgroup interactions at each residue of the Kir2.2 channel. (*A*) Structuring shows PIP_2_ interactions in the *PM* simulation, alongside a bar graph showing the interaction frequencies of those residues with >2.5% of the total PIP_2_ headgroup interactions. (*B*) Structures show PS headgroup interactions in the *PM* (*Left*) and the *No PIP*_*2*_ (*Right*) simulations, alongside a bar graph showing the interaction frequencies of those residues with >2.5% of the total PS headgroup interactions. The definition of lipid headgroups is detailed in *Methods*.

We examined in more detail the headgroup interaction sites of PIP_2_ and PS (Fig. 3). The PIP_2_ headgroup interacts with residues R78, R186, K188, and K189 of the primary interaction site. Further, PIP_2_ molecules clustered around Kir2.2 also can interact at the putative secondary interaction site residue K220. However, interaction is not as strong at K62, which is the secondary anionic lipid interaction site residue that appears to be more important from previous mutagenesis experiments (35, 36). PIP_2_ headgroups can interact with protein residues on the cytoplasmic domain that are far from the membrane domain, which may be due to membrane undulations in the large membrane simulations (see, e.g., *SI Appendix*, Fig. S7). The PS headgroup interacts at K62 and K220, in agreement with experimental data (34–36), with a preference for interaction at K62, especially when PIP_2_ is present. However, PS can also interact with residues of the primary PIP_2_ interaction site (residues R78, R80, K183, R186, and K189).

### Distinct Interaction Site Analysis for PIP_2_ and PS Reveals Many Sites with Diverse Kinetic Profiles.

Lipid headgroup interaction hotspots (shown in Figs. 2 and 3) do not show which residues interact simultaneously to form a lipid headgroup interaction site (i.e., a distinct group of residues that interact simultaneously with a specific lipid headgroup), although it is clear that the surface of the Kir2.2 molecule could present multiple interaction sites. In order to identify which clusters of interacting residues form separate sites, a graph-theoretic approach is useful: the residues form the nodes of the graph and the graph edges that link residues are weighted according to how frequently they cointeract with a single lipid molecule. Using an analysis based on “community” formation (25), residues can be grouped into clusters that form separate binding sites. This approach has been used recently to identify Chol interaction sites on Kir2.2 channels (26).

In order to understand the kinetics of lipid interactions at the surface of Kir2.2 channels, the interaction time of each lipid headgroup at the surface is required. The residence time is a measure that has been used previously (15, 19–21, 48). We calculated residence times for the distinct interaction sites identified using the analysis described above.

**Fig. 4.**
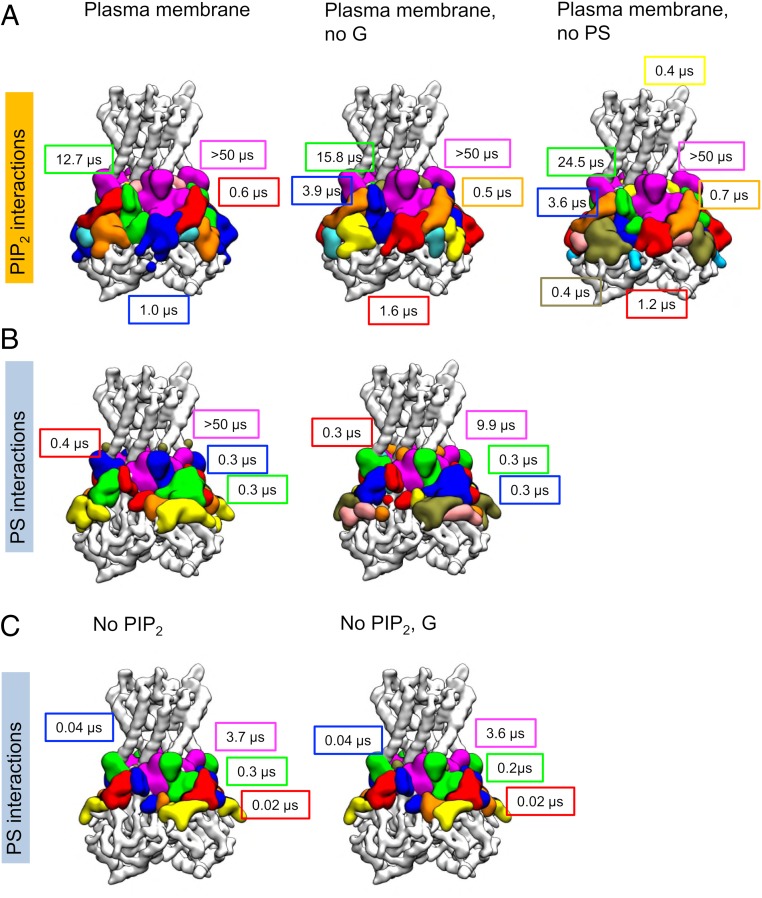
PIP_2_ and PS headgroup interaction sites and corresponding residence times. Lipid headgroup interaction sites were identified by network analysis of residues that simultaneously interact with the headgroup of an individual lipid molecule (see *Methods* for details). (*A*) PIP_2_ headgroup interaction sites for the simulations of nine Kir2.2 channels embedded in (left to right): a PM (simulation *PM_s*; see *SI Appendix*, Table S1 for details), a PM with glycolipids removed (simulation *No G_s*), and a PM without PS (simulation *No PS_s*). (*B*) PS headgroup interaction sites for the simulations *PM_s* (*Left*) and *No G_s* (*Right*). (*C*) PS headgroup interaction sites in the *No PIP*_*2*_*_s* and *No PIP*_*2*_ *G_s* (i.e., with PIP_2_ and GM3 removed) simulations. The lipid headgroup interaction sites are colored according to the residence time, from longest residence time to shortest: magenta, green, blue, red, orange, yellow, and tan. Interaction sites are labeled with the residence time in the corresponding colors. Interaction sites with residence times <0.1 µs are not labeled (except for the red and blue interaction sites in *C*, whose equivalents in *B* had been >0.1 µs). Residence times are quoted to the nearest 0.1 µs (apart from the two exceptions just noted). Residues that make up each interaction site are detailed in *SI Appendix*, Table S2, which also details the error of fitting for residence times. The lipid composition of each simulation is detailed in *SI Appendix*, Table S1.

Using this approach, multiple, distinct interaction sites were identified for PIP_2_ and PS headgroups at the Kir2.2 channel surface (Fig. 4 and *SI Appendix*, Table S2), and by calculating the residence time at each interaction site we showed that PIP_2_ and PS interact at multiple sites with diverse kinetic profiles.

**Fig. 5.**
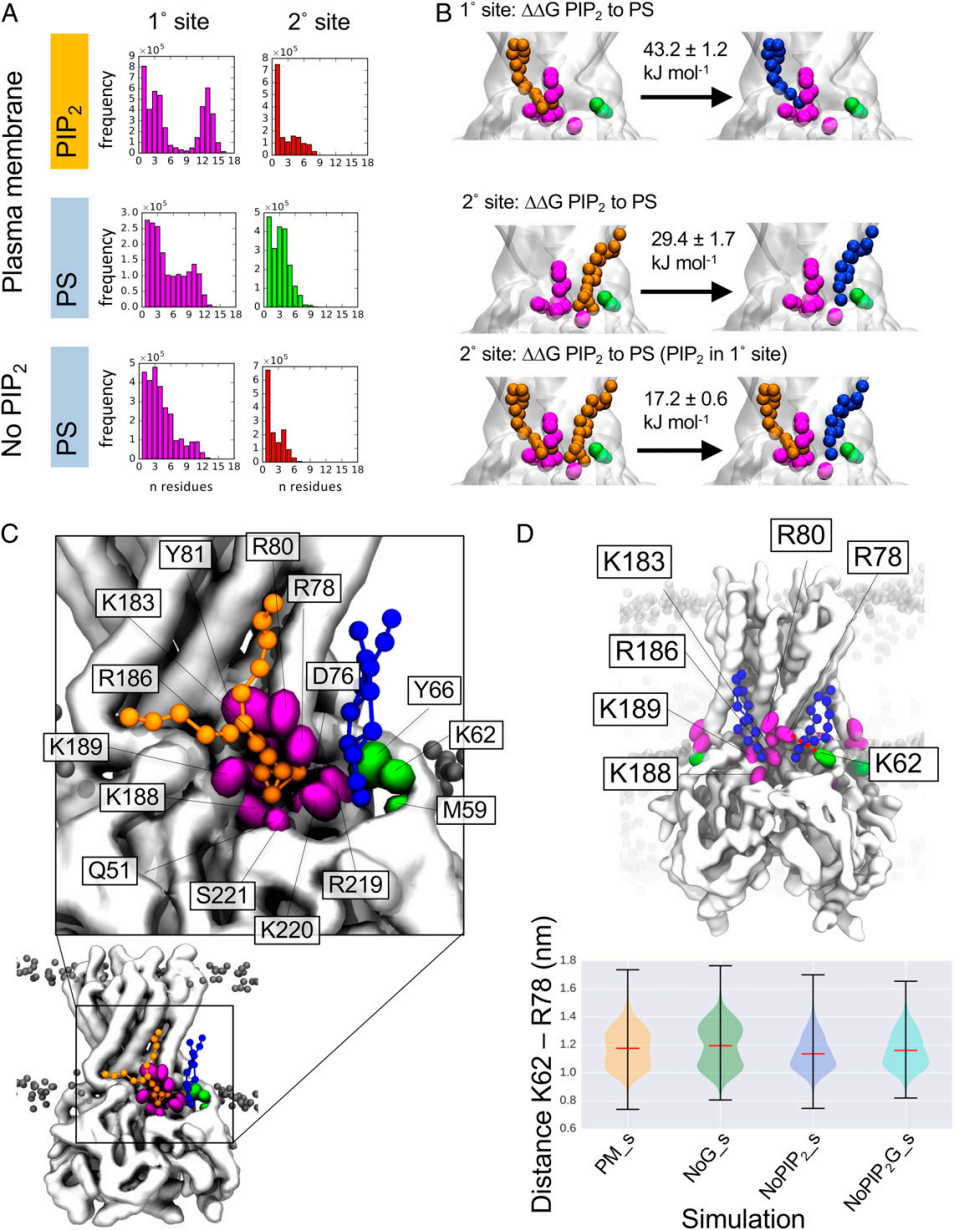
Characterization of PIP_2_ and PS binding at primary and secondary interaction sites. (*A*) The number of contacts made simultaneously by a single lipid molecule when interacting at the primary (*Left*) or secondary (*Right*) interaction sites. The number of protein residue contacts at each interaction site made by PIP_2_ in the PM (*PM_s*) simulations is shown in the top row, PS interactions in the PM (*PM_s*) simulations is shown in the middle row, and PS interactions in the *No PIP*_*2*_*_s* simulations on the bottom row. Sites are colored according to the color scheme in Fig. 4. The number of contacts made by PIP_2_ and PS for all interaction sites identified in Fig. 4 is shown in full in *SI Appendix*, Fig. S8. (*B*) FEP calculations of the ∆∆G of PIP_2_ vs. PS interacting at the primary site (*Top*), the secondary site when PIP_2_ is not bound at the primary site (*Middle*), and the secondary site when PIP_2_ is bound at the primary interaction site (*Bottom*). (*C*) Snapshots demonstrate typical binding of PIP_2_ (orange ball and stick) in the primary interaction site and PS (blue ball and sticks) at the secondary interaction site during the course of the simulation. The 12 residues interacting with the PIP_2_ headgroup in this snapshot are shown with their side chains in magenta, and 3 residues interacting with the PS headgroup are shown with their side chains in green. Only the backbone of D76 is interacting with PIP_2_; otherwise, all lipid headgroup interactions are with residue side chains. The protein backbone is shown in white and phosphate beads of other lipids in the simulation are shown as gray spheres. (*D*) Analysis of the distance between R78 and K62 (shown as a red dotted line on the structure). The structure shows a snapshot of two PS molecules (blue ball and stick) interacting at the surface of Kir2.2 channel. The protein backbone is shown in white and residues of the primary and secondary sites are shown with their side chains in magenta and green, respectively. Phosphate headgroups of other lipids in the bilayer are shown as pale gray spheres. The violin plots show the distance between K62 and R78 for the *PM_s* (yellow), *No G_s* (green), *No PIP*_*2*_*_s* (blue), and *No PIP*_*2*_*G_s* (cyan) simulations. *PM_s* and *No G_s* both contain PIP_2_, while *No PIP*_*2*_*_s* and *No PIP*_*2*_*G_s* do not; for full details of the lipid composition see *Methods*.

To further characterize PIP_2_ and PS headgroup interactions at the each site, we analyzed the number of protein residues that a single lipid headgroup interacts with simultaneously when at a given interaction site (Fig. 5*A* and *SI Appendix*, Fig. S8) and performed FEP calculations (46) for PIP_2_ compared to PS interactions at the primary and secondary interaction sites (Fig. 5*B*).

### PIP_2_ has Most Long-Lived Interaction at the Primary Interaction Site.

By far the most long-lived interaction site for the PIP_2_ headgroup coincided with the experimentally determined primary PIP_2_ interaction site. PIP_2_ headgroups also interacted with Kir2.2 channels at several other interaction sites, all of which were also present in simulations from which where GM3 was removed (comparing left and middle columns in Fig. 4*A*). GM3 should not affect PIP_2_ headgroup interactions, since GM3 is in the outer leaflet of the membrane, providing evidence that the identified interaction sites are reproducible.

Analysis of the number of contacts made simultaneously by any single PIP_2_ headgroup when interacting at the primary interaction site (Fig. 5 *A*, *Top Left*) shows that there are a population of PIP_2_ molecules that frequently interact with only one residue (most often Q51, which has the highest interaction frequency; Fig. 3) and a population that form simultaneous interactions with many more residues (9 to 15 residues), which constitute a more stable interaction. This result is also observed in simulations where GM3 is present (*SI Appendix*, Fig. S8). A snapshot of PIP_2_ interacting at this site, with the PIP_2_ headgroup in contact with 12 residues of Kir2.2, is shown in Fig. 5*C*.

All other PIP_2_ headgroup interaction sites had much lower residence times than that of the primary PIP_2_ interaction site and made many fewer simultaneous contacts (maximum 10) than at the primary site (maximum 16; *SI Appendix*, Fig. S8, *Top*). Even so, there was some heterogeneity between the remaining interaction sites. PIP_2_ headgroups interacted at a site containing residues Q64, R65, I67, and A68 of the slide helix and residues K48, N49, G50, A191–H198 and Q311–A312, S314, and Y316 of the cytoplasmic domain. This interaction site had the second-highest residence time (12.7 ± 0.6 µs in the PM simulations). At this site a population of PIP_2_ headgroups interacted with five to seven residues simultaneously (*SI Appendix*, Fig. S8). This site was split in two and had residence times of 15.8 ± 1.1 µs and 3.9 ± 0.2 µs when GM3 was not included. A third distinct site was identified further down on the cytoplasmic domain (containing residues 41 to 44, 47, 53 to 54, 315, and 335 to 336), with a residence time in order of 1 to 2 µs. PIP_2_ headgroup interactions at the secondary anionic lipid interaction site were on the submicrosecond (i.e., hundreds of nanoseconds) timescale, indicating a less-tight PIP_2_ headgroup interaction at this secondary site than at the primary interaction site. Together with the lower interaction frequency of PIP_2_ at this site (Fig. 3), this indicates that PIP_2_ interacts relatively little at the secondary anionic lipid interaction site. Analysis of the number of simultaneous contacts that the PIP_2_ headgroup could make at this site showed that most frequently PIP_2_ headgroups interacted at this site via only one residue, while a small proportion contacted three to nine residues (Fig. 5*A*). A typical snapshot of the latter type of interaction is shown in *SI Appendix*, Fig. S9*A*.

### PS Can Interact at Primary and Secondary Interaction Sites.

PS headgroup interaction sites in the PM simulations (and in PM without GM3 present) were different from those of PIP_2_ headgroups, further highlighting that PIP_2_ and PS headgroups occupy distinct sites on the surface of Kir2.2 (Fig. 4*B*). The interaction of the PS headgroup at the primary site was more restricted in the number of interaction residues (*SI Appendix*, Table S2) and made fewer simultaneous protein contacts (Fig. 5*A*), likely because PS has a smaller headgroup. The site shifted toward residues A69, M70, T71, and T72, which sit in the kink of the slide helix, with backbone atoms facing the primary site (but not interacting with the PIP_2_ headgroup in either the crystallographic or simulation PIP_2_ binding sites). Intriguingly, in some cases PS was able to interact at the primary site along with PIP_2_, and it appears that PS can be “trapped” behind a PIP_2_ molecule (see snapshot in *SI Appendix*, Fig. S9*B*). Accordingly, the residence time of this interaction decreases from ≥10 µs to 4 µs (to the nearest microsecond) when PIP_2_ is absent from the membrane (Fig. 5*C*). The interaction time of PIP_2_ is not affected; however, this interaction may explain the much-increased residence time of PS at the primary interaction site when PIP_2_ is present.

PS headgroup interactions at the N terminal of the first TM (transmembrane) helix (R78 and R80) formed a separate interaction site, whereas for PIP_2_ these residues are part of the primary interaction site. The PS headgroup interaction site containing residues R78 and R80 had a residence time of 0.25 ± 0.02 µs.

A third interaction site encompassed residues K62 and K220, which had previously been determined as the secondary anionic lipid site in experiment. This interaction site also has an intermediate residence time of 0.28 ± 0.02 µs. At this site, coinciding with the experimentally determined secondary interaction site, there was a population of PS molecules that interacted at the site via only one residue, and a second population that interacted with two to seven residues (a snapshot of a PS interaction typical of the latter population is shown in Fig. 5*C*).

Like PIP_2_, PS headgroups also formed further interactions on the other side of the slide helix (containing residues of the slide helix 64, 65, 67, and 68 and cytoplasmic residues 48, 49, and 191). PS headgroups made very transient interactions with Kir2.2 channels further “out” on the cytoplasmic domain—these may have occurred when there were membrane undulations (*SI Appendix*, Fig. S7).

In the same way as PIP_2_, the PS headgroup also had longer interaction times at the primary interaction site than at the secondary interaction site, suggesting that PS headgroups can interact at the primary site but that PIP_2_ outcompetes PS. PS headgroup interaction sites were similar when GM3 was removed; the most marked difference was the residence time at the primary interaction site, indicating that the high residence time in the PM simulation may have been caused by only a few long interactions.

### PIP_2_ Outcompetes the Secondary Anionic Lipid (PS) at the Primary Interaction Site.

By comparing the behavior of lipids in simulations containing diverse membrane compositions (Figs. 3 and 4 and *SI Appendix*, Fig. S5) one can explore the interplay of different lipids at the surface of Kir2.2 channels. Thus, by comparing PIP_2_ headgroup interaction sites in simulations with and without PS (Fig. 4*A* and *SI Appendix*, Table S2) it is evident that PS does not alter the residues involved in the primary interaction site or the residence time. The position and corresponding residence time of the PIP_2_ headgroup interaction at the secondary interaction site were also not affected by the presence of PS (Fig. 4*A*). This underlines that the experimentally observed effect (34–36) that the secondary anionic lipid (PS) has on increased channel activity is only via the secondary anionic lipid site.

In order to explore further the relative interaction free energies of PIP_2_ and PS at the primary and secondary interaction sites, free energy perturbation calculations were performed (Fig. 5*B*) in which we calculated the *∆∆*G for replacement of bound PIP_2_ by bound PS. The data reveal that PIP_2_ binds much more strongly to the primary site than PS (∆∆G of 43.2 ± 1.2 kJ/mol), meaning that PIP_2_ would outcompete PS at the primary site. This can be rationalized by the number of contacts that the PIP_2_ headgroup is able to form at the site in comparison to those of the PS headgroup (Fig. 5*A*). The PIP_2_ headgroup can form interactions via all three anionic phosphate moieties with the large number of positively charged residues in the interaction site (R78, R80, K183, R186, K188, and K189).

PIP_2_ interaction at this site is functionally important because PIP_2_ is understood to open the channel by favoring the conformation of Kir2.2 in which the cytoplasmic and transmembrane domains are brought together (33). PIP_2_ can achieve this by binding to multiple residues in both the cytoplasmic and transmembrane region; the headgroup of PS cannot interact so extensively and therefore would be unlikely to bring about similar conformational change.

In order to examine the effect of lipid interactions on protein conformation, root-mean-squared fluctuation calculations were performed. Although MARTINI does not allow for changes in protein secondary structure, root-mean-squared fluctuations of the protein indicate that when PIP_2_ is present, linker regions on both N- and C-terminal ends of the transmembrane domain (i.e., the slide helix and around residues 188 to 189) are more stable than when PIP_2_ is not present (*SI Appendix*, Fig. S10). This suggests that PIP_2_ stabilizes the linkers between the cytoplasmic domain and the transmembrane domain, both at the N- and C-terminal ends of the transmembrane domain, which is in agreement with the effect of PIP_2_ on channel structure as seen in crystal structures.

### PS Interaction at the Secondary Site Is More Favorable when PIP_2_ Interacts at the Primary Site.

While PIP_2_ headgroup interactions seem to be impacted relatively little by PS, it appears there is some interplay in terms of effect of PIP_2_ on PS headgroup interactions (Fig. 4 *B* and *C* and *SI Appendix*, Table S2). Surprisingly, it appears that the PS headgroup interaction at the secondary anionic lipid site was affected by the presence of PIP_2_. When PIP_2_ was absent from the membrane, the PS headgroup interaction at K62 involved fewer residues and residence times were an order of magnitude lower: When PIP_2_ was present in the membrane, the site included residues E55 to R65, Y66, L218 to K220, and Y342-S343, but when PIP_2_ was absent the site did not include residues Y66 and R219 to K220, which instead became part of the interaction site at residues R78 and R80 (*SI Appendix*, Table S2). The residence time for the site at R78 and R80 is not diminished when PIP_2_ is not present, but the secondary anionic lipid site (i.e., the site including residue K62) has a residence time of 0.28 ± 0.02 µs when PIP_2_ was present, diminishing to 0.02 µs when PIP_2_ was absent.

FEP calculations also show that when PIP_2_ is not interacting at the primary site, the secondary site has a ∆∆G of 29.4 kJ/mol, that is, a strong preference for PIP_2_ (albeit much less so than at the primary site); when PIP_2_ is present in the primary site the ∆∆G is 17.2 kJ/mol, i.e., far less strong preference for PIP_2_ over PS. That is, PIP_2_ interaction at the primary site renders the interaction of PS at the secondary site more favorable.

The effect of PIP_2_ at the primary site on interactions at the secondary site may be due to the increased propensity for the positively charged side chain of R78 to be orientated toward K62 when PIP_2_ is not interacting at the primary site (Fig. 5*D*). In simulations where PIP_2_ is present (*PM_s* and *No G_s*), the distance between R78 and K62 has a bimodal distribution (the conformation corresponding to the larger distance is seen in snapshot in Fig. 5*C*; the shorter distance is as seen in snapshot, Fig. 5*D*). When PIP_2_ is not present (simulations *No PIP*_*2*_*_s* and *No PIP*_*2*_ *G_s*), the distance distribution shifts, such that R78 and K62 side chains are more likely to be close together (as in the snapshot, Fig. 5*D*). The PS headgroup at the secondary site (at K62) is more likely therefore to move between the site at K62 and the site at R78, perhaps explaining why the residence time of the secondary interaction site is so much lower when PIP_2_ is not present.

The interaction between PS and K62 appears to be functionally important—mutagenesis and electrophysiological studies have shown that K62 tethering to the membrane recovers the effect of anionic lipid interactions, indicating that it is the tethering role which is important. The current study shows that before PIP_2_ has started to interact at the primary site this tethering via K62 is less likely, because PS is more likely to bind at the site containing R78 and R80.

As discussed above, the free energy of binding of PS compared to that of PIP_2_ is positive, indicating that PIP_2_ is the preferable lipid even at this secondary site, regardless of whether PIP_2_ also occupies the primary site. However, it is evident from FEP calculations and the residence times that a PIP_2_ interaction is more likely to occur at the primary site than at the secondary site, because the PIP_2_ headgroup cannot form as many contacts at the secondary site as it can at the primary site, and in particular there are fewer positively charged residues in the interaction site (*SI Appendix*, Fig. S9*A*). The more favorable free energy of binding for PIP_2_ even at the secondary site suggests that the interaction of PS at this site is dependent on its concentration relative to PIP_2_.

PS interacts at the secondary site, and aside from the primary interaction site this is the site where PS appears to form the most contacts with the protein (*SI Appendix*, Fig. S8).

### There Is Little Interplay between Anionic Lipids and Cholesterol.

PIP_2_ and PS headgroup interactions are not affected by Chol (*SI Appendix*, Fig. S5) and neither do Chol interactions appear to be affected by the presence or absence of PIP_2_ or PS (*SI Appendix*, Fig. S5). There is a slight decrease in the Chol residence times in simulations where PIP_2_ are not present (*SI Appendix*, Table S3). However, all Chol residence times are low, in the order 0.8 to 1 µs (for comparison, PIP_2_ interactions at the primary site are greater than 50 µs) and the relatively small variation in Chol residence times may not bear great functional significance. There is no noticeable interplay between GM3 and Chol (*SI Appendix*, Fig. S5).

## Discussion

The coarse-grained simulations of Kir2.2 channels in membranes containing multiple lipid species allowed us to observe the complex interplay of PIP_2_, PS, and other lipids as they interacted at the surface of the channel. Multiple lipid species are known to interact and affect the activity of Kir2.2, but previous simulation studies have focused on a single lipid type [e.g., just PIP_2_ (16, 17) or just Chol (26)], and to the best of our knowledge there have been no simulation studies investigating lipid interactions containing the secondary anionic lipid, either as a single lipid species or in a complex mixture. Using the graph-theoretic approach adopted by ref. 26, we were able to identify distinct binding sites of PIP_2_ and PS headgroups on the surface of Kir2.2. We characterized the distinct interaction sites by calculating lipid headgroup residence times and the number of contacts made in each site, thereby assessing how stable the lipid headgroup interaction was at each site. FEP calculations were performed to quantify the relative free energies of binding of PIP_2_ and PS at the primary and secondary interaction sites.

These simulations and their analysis allowed us to show that the tightest interaction site for PIP_2_ is, as expected, at the primary interaction site. PS can interact at the primary site and will replace PIP_2_ when PIP_2_ is absent, but PS cannot form the same number of contacts with as many positively charged residues as PIP_2_ and FEP calculations show that PIP_2_ has a much more favorable free energy of binding, and thus the PS headgroup is outcompeted by the PIP_2_ headgroup at the primary site. Functionally the interaction with PS is less favorable, since the PS headgroup cannot link the cytoplasmic and transmembrane domains as PIP_2_ is able to do.

The simulations also identify the experimentally characterized secondary anionic lipid site at residue K62. FEP calculations showed that, even at this site, PIP_2_ binds more favorably. However, we have shown that any nearby PIP_2_ molecule is far more likely to bind to the primary site, leaving the secondary site free for PS binding, as long as the local concentration of PIP_2_ is low enough. This agrees well with experimental data (34), which show that the secondary anionic lipid effect on channel opening occurs only at lower PIP_2_ concentrations (less than 10% PIP_2_); in our PM simulations PIP_2_ comprised 5% of the total lipid composition.

By combining equilibrium simulations of Kir2.2 in a variety of lipid mixtures with FEP calculations, we show that there is interplay between PIP_2_ and PS at the secondary interaction site: when PIP_2_ interacts at the primary site, PS interactions at the secondary site become more favorable. Residence times of PS at this site show that PS interacts more tightly when PIP_2_ is present, and FEP calculations further showed that the presence of PIP_2_ in the primary site decreased preference for PIP_2_ over PS at the secondary site. Interplay between PIP_2_ and PS has been indicated previously by docking studies (35) and suggests that interaction of PS at the secondary site principally occurs subsequent to PIP_2_ binding at the primary site. Here we show that the tighter interaction of PS at this secondary site may be due to the rearrangement of positively charged residue R78 at the primary site, once PIP_2_ is interacting.

PIP_2_ interactions were not affected by the absence of PS, confirming that the enhancement of channel activity by a secondary anionic lipid is not via interplay with PIP_2_ at the classical interaction site, but only by direct interactions at the secondary interaction site.

### Further Considerations.

The primary PIP_2_ interaction site identified in the simulations here is slightly extended compared to the crystal structures (33, 36). The graph-theoretic method used here to identify interaction sites grouped residues that frequently cointeracted with one PIP_2_ molecule, but PIP_2_ headgroups interacting at this site were not interacting with all residues in the site simultaneously (Fig. 5*A*). The enlarged site may represent slight movement of PIP_2_ headgroup in the interaction site, or may be due to different PIP_2_ molecules adopting a range of interaction positions with different copies of Kir2.2 present in the simulation, as a result of the complex lipid mixture (cf. the variation in the number of simultaneous contacts formed by the PIP_2_ headgroup in the primary site, Fig. 5*A*). This also may be due to more than one PIP_2_ interacting close to the primary interaction site, thereby extending the site; we note that in simulations containing only a single copy of Kir2.2 and a low number PIP_2_ molecules, PIP_2_ occupied the crystal structure interaction sites (17). In the current study, we have based the concentration of PIP_2_ on PM lipidomic studies (49); it could be an interesting direction of further study to perform simulations involving lower concentrations of PIP_2_ in a mixed lipid bilayer, to account for possible heterogeneity within the PM of PIP_2_ concentration.

In agreement with previous results (35, 36), K62 is identified as the key secondary interaction site residue in this study. K220 has also been implicated as a residue involved in the secondary anionic lipid interaction, albeit with less effect on channel activation [K220C mutations showed greater maximal activity than K62C mutations, and tethering of K62C to the membrane via decyl modification to the cysteine displayed greater restoration of activity than channels containing K220C decyl modifications (35)]. In our simulations, K220 forms part of the primary interaction site with PIP_2_. It is possible that K220 transiently interacts with PS, leading to weak stabilization, thus leading to some bulk anionic lipid-dependent effect on channel activation when mutated to cysteine.

We note that lipids used in experiments to identify K62 as the key residue in the secondary interaction site are PG and/or PA and not necessarily PS, but here we use PS, since it is the second-most-abundant anionic lipid present in the mammalian PM. In early studies identifying the anionic lipid dependence of channel activation, PS was used and did not show any difference from PA and PG (34).

To further assess the interaction of PS and the impact of PIP_2_, it would in principle be useful to be able to perform simulations of the pre-PIP_2_-bound state of the channel. However, K62 is at the start of a disordered region of the PPA-bound structure (Protein Data Bank [PDB] ID code 3SPC), and accurate modeling of disordered regions remains a challenge; therefore, it would be difficult to draw firm conclusions from simulations based on such a structure + model.

As identified previously (44), Chol interacts at distinct sites compared to PIP_2_ and PS headgroups, and we did not observe a shift in interaction site of either PIP_2_ or PS on removal of Chol from the membrane. There was a very slight decrease in Chol interaction times in simulations where PIP_2_ was not present vs. where PIP_2_ was present; however, this decrease was small. This indicates that inhibition of Kir2.2 by Chol operates independently from channel opening by PIP_2_ and other anionic lipids.

### Possible Limitations.

The use of the graph-theoretic and residence-time analysis allows for multiple lipids to be observed and avoids predefining an interaction site to explore. However, residence times longer than the simulation time cannot be inferred, and this limits the conclusions that can be drawn regarding the effect of different lipid types on the most long-lived PIP_2_ interactions (which all occur at the crystallographic interaction site). Enhanced sampling methods and/or Markov state models could be used but with more than two lipid types they could be challenging to implement.

Simulations using the MARTINI coarse-grained approach have provided valuable insights into the behavior of lipids around Kir channels (16) and around other channel and receptor proteins (14). However, the MARTINI model fixes the secondary structure of the protein, which limits the opportunity to probe the effects of lipid interactions of conformational changes underlying channel gating would proceed, and thus on how lipid complexity might influence channel activity. Root-mean-squared fluctuations of the protein in simulations with and without PIP_2_ are in agreement with the proposed effect of PIP_2_ on channel opening; however, further work is required to understand how the interplay of PIP_2_ and PS interactions might affect channel gating, especially as progress has been made recently in understanding the effect of phospholipids on gating of Kir2.2 channels via a combined structural and computational approach (50).

It is also worth noting that within MARTINI electrostatic interactions are represented using a shifted function with a cutoff at 1.2 nm and with screening included implicitly, rather than employing the particle mesh Ewald approximation. This may mean that long-range electrostatic interactions are underestimated. Since the lipids of principal interest in this study are anionic, interacting with the protein via electrostatic interactions, this could present a further limitation. However, the interactions do take place over relatively short distances (less than 1.2 nm). The behavior of PIP_2_ headgroups could be altered if, for instance, a polarizable coarse-grained water model (51) were to be used (and this would be an interesting direction for further study). Nevertheless, we also note that MARTINI model for anionic lipids has been shown to be in good agreement with experimental data in a number of examples (3, 16, 17, 52).

Chol interactions do not appear to be impacted by the presence of PIP_2_, as previously indicated by Rosenhouse-Dantsker et al. (44), who have shown that PIP_2_ interactions sites (and indeed the secondary anionic lipid interaction site) do not overlap with the Chol interaction sites. It is possible that ordered lipid nanodomains (enriched in Chol and sphingolipids) (53) may modulate channel function and interactions (54, 55). However, in our current simulations we did not include saturated phospholipids that would have been needed to colocalize with Chol to form such nanodomains. This could be an interesting direction for future studies.

## Conclusions

The lipid compositional complexity of cell membranes can have a profound influence on the biological functions of ion channels. The current study has employed large coarse-grained MD simulations to probe the effect of lipid complexity on Kir2.2, a membrane protein with multiple, well-characterized lipid interactions. We analyzed lipid interactions of Kir2.2 in a membrane containing PIP_2_ along with a secondary anionic lipid (PS), Chol, and other lipid species including gangliosides at physiological concentrations. PIP_2_, the lipid required to activate the channel, interacts at crystallographically identified primary interaction sites, and the secondary anionic lipid, PS, interacts at a previously identified secondary anionic lipid interaction site. PS can also interact at the primary interaction site but is outcompeted by PIP_2_. At the secondary interaction site, PIP_2_ still has a more favorable interaction free energy than PS, but is more likely to interact at other sites; thus, interaction of PS at the secondary site is dependent on the concentration of PIP_2_. Further, we reveal an interplay in lipid interaction: Once PIP_2_ is interacting at the primary site, interactions of PS at the secondary interaction site become more energetically favorable, rebalancing the likelihood of PS vs. PIP_2_ interaction at the secondary site. This study demonstrates the nuanced competition between distinct protein–lipid interaction sites and highlights the need to study lipid interactions in membranes with physiological lipid compositions.

## Methods

### System Setup and Simulations Performed.

The simulations performed are summarized in *SI Appendix*, Table S1. The Kir2.2 channel crystal structure (PDB ID code 3SPI; ref. 33) was converted to a coarse-grained representation using the martinize.py script (version 2.4; http://md.chem.rug.nl/index.php/tools2/proteins-and-bilayers) with the MARTINI2.2 force field (12, 13) and the ElNeDyn elastic network model (56). A single coarse-grained model of Kir2.2 was embedded into a PC bilayer by allowing lipids to self-assemble around the protein in a short simulation (57). The PC membrane was subsequently converted into a membrane of the desired lipid composition using an in-house exchange lipid methodology (58). The Kir2.2 channel in a mixed lipid membrane was then equilibrated for 100 ns prior to being tessellated, using the GROMACS tool genconf, to form 12 × 12 and 3 × 3 grids. The resulting systems contained either 144 or 9 Kir2.2 channels, ∼55,000 (12 × 12 systems) or ∼3,500 (3 × 3 systems) lipids, and were solvated using the standard MARTINI water model and neutralized to a 0.15 M NaCl concentration (see *SI Appendix*, Table S1 for details).

Lipid bilayers were modeled with a mixture of the most abundant lipids present in mammalian cell PM (49, 59, 60), with lipids distributed asymmetrically between the inner and outer leaflets, as previously described (58, 61). In the PM model, which is the most complex presented here, the outer leaflet contained PC/PE/sphingolipid (Sph)/ganglioside (GM3)/Chol lipids in a ratio of 40:10:15:10:25, while the inner leaflet contained PC/PE/PS/PIP_2_/Chol in a ratio of 10:40:15:10:25. PC, PE, and PS were modeled with 1-palmitoyl-2-oleoyl lipid tails. To generate the *No PIP*_*2*_ simulation, PIP_2_ was removed from the PM model by substitution with PE, as these two species clustered in areas of higher membrane curvature in previous PM simulations (58). In simulations where lipid complexity was further reduced, the proportions of the PM simulation were maintained for all remaining lipid species (*SI Appendix*, Table S1). Sph and GM3 were modeled with a monounsaturated ceramide tail. Lipids were modeled using the MARTINI 2.1 parameters, except for PIP_2,_ modeled as described previously (16), and GM3 (58); the MARTINI beads used for PIP_2_ and PS are shown in *SI Appendix*, Fig. S1*B*.

### Simulation Parameters.

All equilibrium simulations were performed using GROMACS 4.6 (http://www.gromacs.org/) and the standard MARTINI protocol. Periodic boundary conditions were applied, and a time step of 20 fs was used in all simulations. The temperature was maintained at 323 K using a Berendsen thermostat (62) and the pressure at 1 bar using a Berendsen barostat. For both the temperature and pressure, a coupling constant of 4 ps was used. In all simulations, the reaction field coulomb type was used with a switching function from 0.0 to 1.2 nm, and the van der Waals interactions were cutoff at 1.2 nm with a switching function applied from 0.9 nm. The LINCS algorithm (63) was used to constrain covalent bonds to their equilibrium values.

### FEP Calculations.

FEP calculations were performed as described in ref. 46. In brief, a pose of Kir2.2 with PIP_2_ bound at both sites 1 and 2 was extracted from the equilibrium data, and the protein and PIP_2_ molecules were built into a 1-palmitoyl-2-oleoyl-*sn*-glycero-3-phosphocholine bilayer using the insane protocol (64). The membrane was allowed to relax around the Kir2.2–PIP_2_ complex using 100 ns of NPT simulation with positional restraints on the protein backbone, and a further 350 ns without. The output of this was used to generate the starting states for the different FEP calculations, by removing a bound PIP_2_, as necessary.

To carry out the FEP calculations, the PO1, PO2, RP1, and RP2 beads were switched into dummy particles with no charge or Lennard-Jones interactions. The RP3 bead was switched from a neutral particle with MARTINI type SP1, into a neutral particle of type P5, as per the MARTINI POPS parameters. Coloumbic interactions were turned off over 10 windows of 250 ns (*n* = 3), followed by Lennard-Jones interactions over a further 10 windows of 250 ns (*n* = 3), employing a soft-core potential. To maintain a neutral system charge, four Na^+^ ions were also decoupled per PIP_2_ molecule. All simulations were run using GROMACS 2019. Free energies were computed using Multistate Bennett Acceptance Ratio, as calculated using alchemical-analysis (65).

### Simulation Analysis.

Radial distribution functions of lipid headgroups around the channels were calculated using the GROMACS tool g_rdf. Root-mean-squared fluctuations of Kir channels were also calculated by a GROMACS tool, g_rmsf. Values were then averaged over time, the nine proteins in the simulation, and over each monomer.

Protein–lipid interactions were identified using in-house scripts, making use of the NumPy (66), MDAnalysis (67, 68), NetworkX (69), and community (https://github.com/taynaud/python-louvain) Python libraries.

Lipid headgroups were considered to be interacting with given protein residues when within 0.65 nm of one another. In these calculations, lipid headgroups were chosen as phosphate plus choline, ethanolamine, or serine moieties for phospholipids PC, PE, and PS, respectively; for PIP_2_ the headgroup was considered as the inositol moiety and all attached phosphate groups; for GM3, the headgroup included all sugar moieties; the Chol headgroup was considered to be the core steroid moiety and associated hydroxyl group, but not the short hydrocarbon tail.

Interaction sites were derived from simulations using a graph-theoretic method that uses a community analysis, as implemented by ref. 26. A graph was constructed with protein residues as nodes. Nodes are joined if a given pair of residues interact simultaneously (i.e., in the same time frame) with an individual lipid molecule. The frequency with which any given pair of residues interacts simultaneously with an individual lipid molecule determines the weight of the edge in the graph. A community algorithm was then used to detect communities within the graph (i.e., sets of highly connected protein residues). The community algorithm used was the best partition function of the community library https://github.com/taynaud/python-louvain, which uses the Louvain method (70) and takes graph edge weights into account. Each community formed was considered an interaction site.

Calculation of residence times has been used in previous simulation studies to look at protein–water (71, 72) and protein–lipid (15, 19–21, 48) interactions. The residence time, *θ*, of a lipid type (e.g., PIP_2_ or PS) is defined as the average time a single lipid headgroup spends continuously interacting with a Kir2.2. Residence times were calculated from the normalized survival time-correlation function, *σ*(*t*):

$$\sigma \left(t\right)=\frac{1}{N_{j}}\;\frac{1}{T-t}\sum_{j=1}^{N_{j}}\sum_{\nu =0}^{T}{ñ}_{j}\left(\nu ,\nu +t\right),$$

where *T* is the total simulation time and *N*_*j*_ is the total number of a lipid type with nonzero interaction time. The function *ρ*_*j*_ (*v*, *v + t*) has value 1 if lipid j continuously interacts with Kir2.2 from time *v* to time *v + t* (inclusive), and 0 otherwise. The value of *v* ran from 0 ns to *T* ns in steps of 1 ns, and the values of *σ*(*t*) were determined for every value of *t* from 0 to *T* ns, at 1-ns intervals. *σ*(*t*) was normalized by dividing by *σ*(0), so that the survival time-correlation function has value 1 at *t* = 0. The normalized time-correlation function was modeled as a sum of exponential functions:

$$\sigma \left(t\right)∼\text{Aexp}\left(\left(-t/\theta_{1}\right)+\text{Bexp}\left(-\text{t}/\theta_{2}\right)\right).$$

The values of *θ*_1_ and *θ*_2_ were determined by fitting the values of *σ*(*t*) to a sum of exponentials. The double exponential assumes that there are two populations of protein–lipid interactions, one of short-lived interactions, where the lipid fails to bind more tightly to the protein, and the other, more long-lived interactions, where the lipid interacts tightly at the specified interaction site. The residence time of the long interaction is reported, since we are more interested in the tight protein–lipid interactions. The errors reported on residence times are the SD error of fitting to the parameter estimate.

In calculating residence times for PIP_2_ and PS interactions, lipid molecules were considered to be interacting at a given interaction site if one or more lipid headgroup particles were within 0.65 nm of at least one residue within the interaction site. In calculating Chol residence times, the distance cutoff was set conservatively at 1 nm (rather than 0.65 nm), since it has been shown that Chol can be mobile even when interacting at specific interaction sites (22, 73, 74). Chol residence times given in *SI Appendix*, Table S3 are for Chol interactions over the entire surface of the channel, rather than at any specific interaction site.

Analysis of number of protein residue contacts simultaneously interacting with a lipid headgroup was performed using the same distance cutoff, that is, less than 0.65 nm between any bead of the residue and any lipid headgroup bead to determine that a given protein residue was in contact with the lipid headgroup. A lipid headgroup was considered to be interacting at an interaction site if it was in contact with at least one protein residue that was part of the interaction site. Interaction sites used were as determined by the graph-theoretic method.

Graphs were plotted using gnuplot 4.6 (http://www.gnuplot.info/) and Matplotlib (75) and molecular visualization used VMD (76).

### Data Availability.

Files (gro) containing the final frames of all trajectories, input tpr files for each simulation, and the mdp file used for all simulations can be found at https://zenodo.org/record/3634884#.XjiLOOunzOR (DOI: 10.5281/zenodo.3634884). Unless otherwise stated, all code used for analysis of simulations can be found at https://github.com/annaduncan/Kir_scripts.

## Supplementary Material

Supplementary File
