# Supplementary material for: Defining how multiple lipid species interact with inward rectifier potassium (Kir2) channels
Source: Proc Natl Acad Sci U S A. 2020 Mar 25;117(14):7803–13. doi: 10.1073/pnas.1918387117 (PMC7149479; doi:10.1073/pnas.1918387117)
Supplement: Supplementary File [file pnas.1918387117.sapp.pdf]

Supporting Information for:

Defining How Multiple Lipid Species Interact with Inward Rectifier Potassium (Kir2) Channels

Anna L. Duncan, Robin A. Corey & Mark S.P. Sansom

Supporting Information: SI Tables S1 to S3 and SI Figures S1 to S10.

Table S1: Simulations Performed

**Simulation**	**Number of proteins**	**Number of lipids**	**Bilayer composition***	**Box length (nm)†**	**Duration (μs)**
Large Systems (~55,000 lipids; ~3.5 M particles)					
PM	144	55,584	PM	137	40
No PIP_2	144	55,584	PM, PIP_2 removed	130	20
PC	144	55,728	PC	145	20
Small Systems (~3500 lipids; ~0.2 M particles)					
PM_s	9	3,474	PM	34	50
No PIP_2_s	9	3,474	PM, PIP_2 removed	33	50
No G_s	9	3,483	PM, GM3 removed	32	50
No PIP_2G_s	9	3,474	PM, PIP_2, GM3 removed	33	50
No Ch_s	9	3,474	PM, Chol removed	35	50
No P2GCh_s	9	3,474	PM, PIP_2, GM3, Chol removed	35	50
PC_s	9	3,483	PC	36	50
No PS_s	9	3,474	PM, PS removed	33	50

* PM = inner: PC:PE:PS:PIP_2:Chol (10:40:15:10:25) & outer: PC:PE:Sph:GM3:Chol (40:10:15:10:25); for the detailed compositions of other bilayers see the Methods section.
† in *xy*; the box height (*z*) was set to approximately 20 nm.

Table S2: PIP$_2$ and PS Interaction Sites and Residence Times (see Figure 4)

**Site**	**Residence time (μs)**	**Residues (residues of the experimentally-identified <u>primary site</u> and *secondary site* are indicated)**
**PM_s: PIP$_2$ interactions**		
Magenta	>50	51, 52, 66, 73, 76, 77, <u>**78**</u>, 79, <u>**80**</u>, 81,<u>**183**</u>,<u>**186**</u>, <u>**188**</u>, <u>**189**</u>, 219, ***220***, 221, 222, 265, 304, 313
Green	12.7 ± 0.6	48, 49, 50, 64, 65, 67, 68, 191, 192, 193, 194, 195, 196, 197, 198, 311, 312, 314, 316
Blue	1.02 ± 0.04	41, 42, 43, 44, 47, 53, 54, 55, 290, 292, 315, 317, 319, 335, 336, 337, 338
Red	0.59 ±0.02	56, 57, 58, 59, 60, 61, ***62***, 63, 218, 334, 339, 340, 341
**No G_s: PIP$_2$ interactions**		
Magenta	>50	51, 66, 73, 76, 77, <u>**78**</u>, 79, <u>**80**</u>, 81, <u>**183**</u>, 184, <u>**186**</u>, <u>**188**</u>, <u>**189**</u>, 219, ***220***, 221, 222, 265, 313
Green	15.81 ±1.14	50, 72, 75, 190, 191, 310, 311, 312
Blue	3.93 ±0.15	48, 49, 64, 65, 67, 68, 192, 193, 194, 195, 196, 197, 198, 314, 316
Red	1.57 ± 0.05	41, 42, 43, 44, 45, 46, 47, 52, 53, 54, 55, 315, 317, 319, 335, 336, 337, 338
Orange	0.46 ± 0.02	56, 57, 58, 59, 60, 61, ***62***, 63, 218, 334, 339, 340, 341
**No PS_s: PIP$_2$ interactions**		
Magenta	>50	51, 52, 66, 73, 76, 77, <u>**78**</u>, 79, <u>**80**</u>, 81, <u>**183**</u>, <u>**186**</u>, <u>**188**</u>, <u>**189**</u>, 219, ***220***, 221, 222, 265, 313
Green	24.53 ± 0.79	48, 49, 50, 64, 65, 67, 68, 72, 190, 191, 192, 310, 311, 312
Blue	3.6 ± 0.11	193, 194, 195, 196, 197, 198, 314, 316
Red	1.15 ± 0.04	41, 42, 43, 44, 45, 46, 47, 53, 54, 315, 317, 335, 336, 337, 338
Orange	0.68 ± 0.03	55, 56, 57, 58, 59, 60, 61, ***62***, 63, 218, 334, 339, 340, 341
Tan	0.41 ± 0.02	199, 320, 325, 328, 342, 343, 344, 345, 346, 347, 348, 349
**PM_s: PS interactions**		
Magenta	>50	68, 69, 70, 71, 72, 79, <u>**183**</u>, 184, 185, <u>**186**</u>, 187, <u>**188**</u>, <u>**189**</u>, 190, 303
Green	0.28 ± 0.02	55, 56, 57, 58, 59, 60, 61, ***62***, 63, 66, 218, 219, ***220***, 342, 343
Blue	0.25 ± 0.02	73, 74, 76, 77, <u>**78**</u>, <u>**80**</u>, 81, 221
Red	0.4 ± 0.01	48, 49, 50, 51, 64, 65, 67, 191, 192
**No G_s: PS interactions**		
Magenta	9.89 ± 0.95	69, 70, 71, 72, 79, <u>**183**</u>, 184, 185, <u>**186**</u>, 187, <u>**188**</u>, <u>**189**</u>, 190, 303, 306
Green	0.30 ±0.02	73, 74, 76, 77, <u>**78**</u>, <u>**80**</u>, 81, 221, 222
Blue	0.30 ± 0.02	55, 56, 57, 58, 59, 60, 61, ***62***, 63, 66, 218, 219, ***220***, 342, 343
Red	0.27 ± 0.02	48, 49, 64, 65, 67, 68, 192, 195
**No PIP$_2$_s: PS interactions**		
Magenta	3.74 ± 0.33	69, 70, 71, 72, 79, <u>**183**</u>, 184, 185, <u>**186**</u>, 187, <u>**188**</u>, <u>**189**</u>, 190, 303, 306
Green	0.26 ± 0.02	66, 73, 76, 77, <u>**78**</u>, <u>**80**</u>, 81, 219, ***220***, 221
Blue	0.04 ± 0.01	48, 49, 64, 65, 67, 68, 192
Red	0.02 ± 0.00	55, 56, 57, 58, 59, 60, 61, ***62***, 63, 218, 342, 343
**No PIP$_2$G_s: PS interactions**		
Magenta	3.59 ± 0.39	69, 70, 71, 72, 79, <u>**183**</u>, 184, 185, <u>**186**</u>, 187, <u>**188**</u>, <u>**189**</u>, 190, 303, 306
Green	0.23 ± 0.02	66, 73, 76, 77, <u>**78**</u>, <u>**80**</u>, 81, 219, ***220***, 221
Blue	0.04 ± 0.01	48, 49, 64, 65, 67, 68, 192
Red	0.02 ± 0.00	55, 56, 57, 58, 59, 60, 61, ***62***, 63, 218, 342, 343

Table S3: Cholesterol residence times

**Simulation**	**Residence time (μs)**
PM_s	1.14 ± 0.08
No PIP_2_s	0.83 ± 0.06
No G_s	0.95 ± 0.07
No PIP_2G_s	0.72 ± 0.05
No PS_s	1.14 ± 0.05

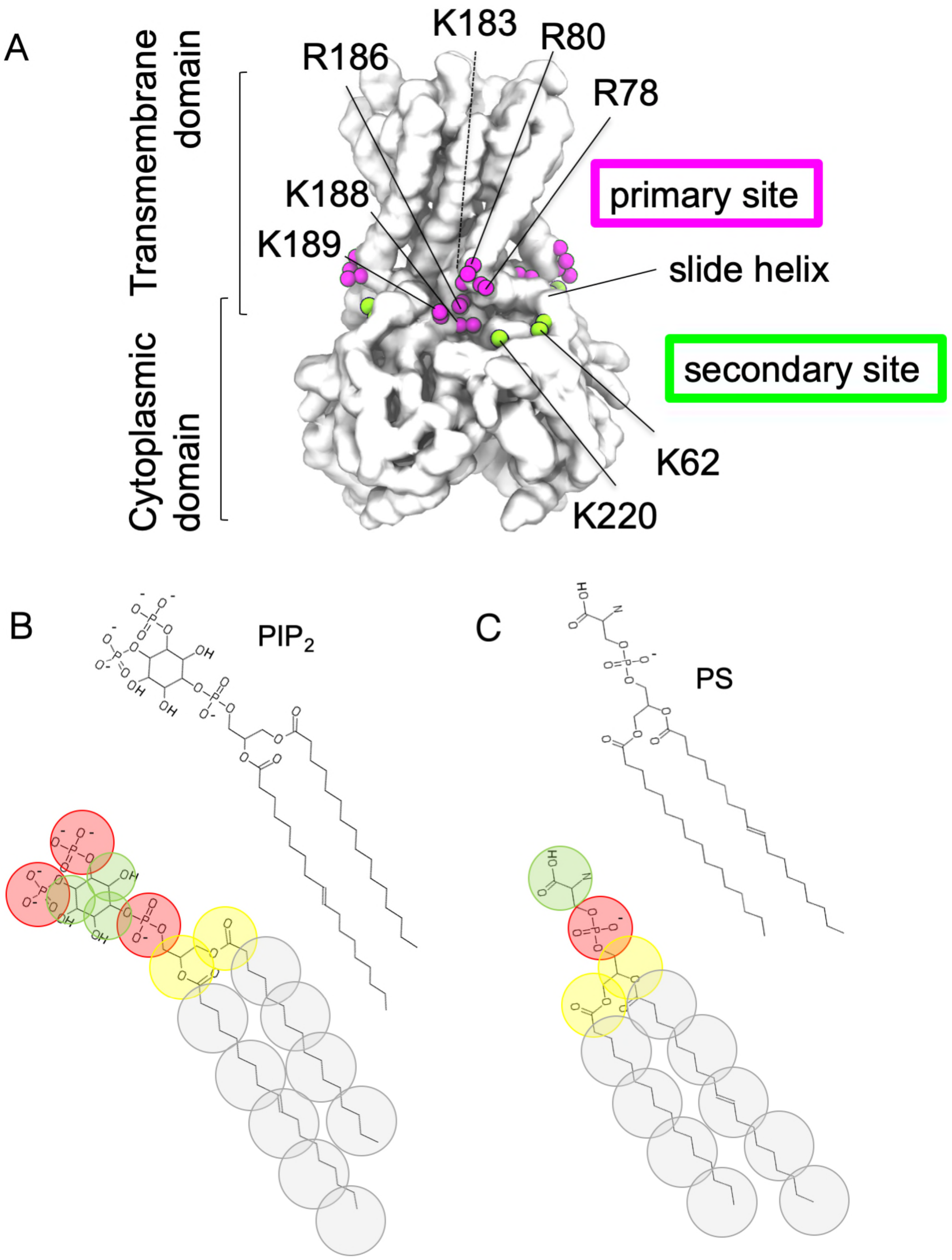

Figure S1. A Residues of the primary (magenta) and secondary (green) lipid interaction sites are shown as spheres on the structure of Kir2.2. **B**. Chemical structure of PI(4,5)P_2 and **C** PS, with their respective MARTINI representations superimposed on chemical structues underneath. In the MARTINI representations, apolar beads are shown in grey, polar beads in green, negatively charged beads in red and nonpolar beads (moieties containing both polar and apolar atomic groups) in yellow.

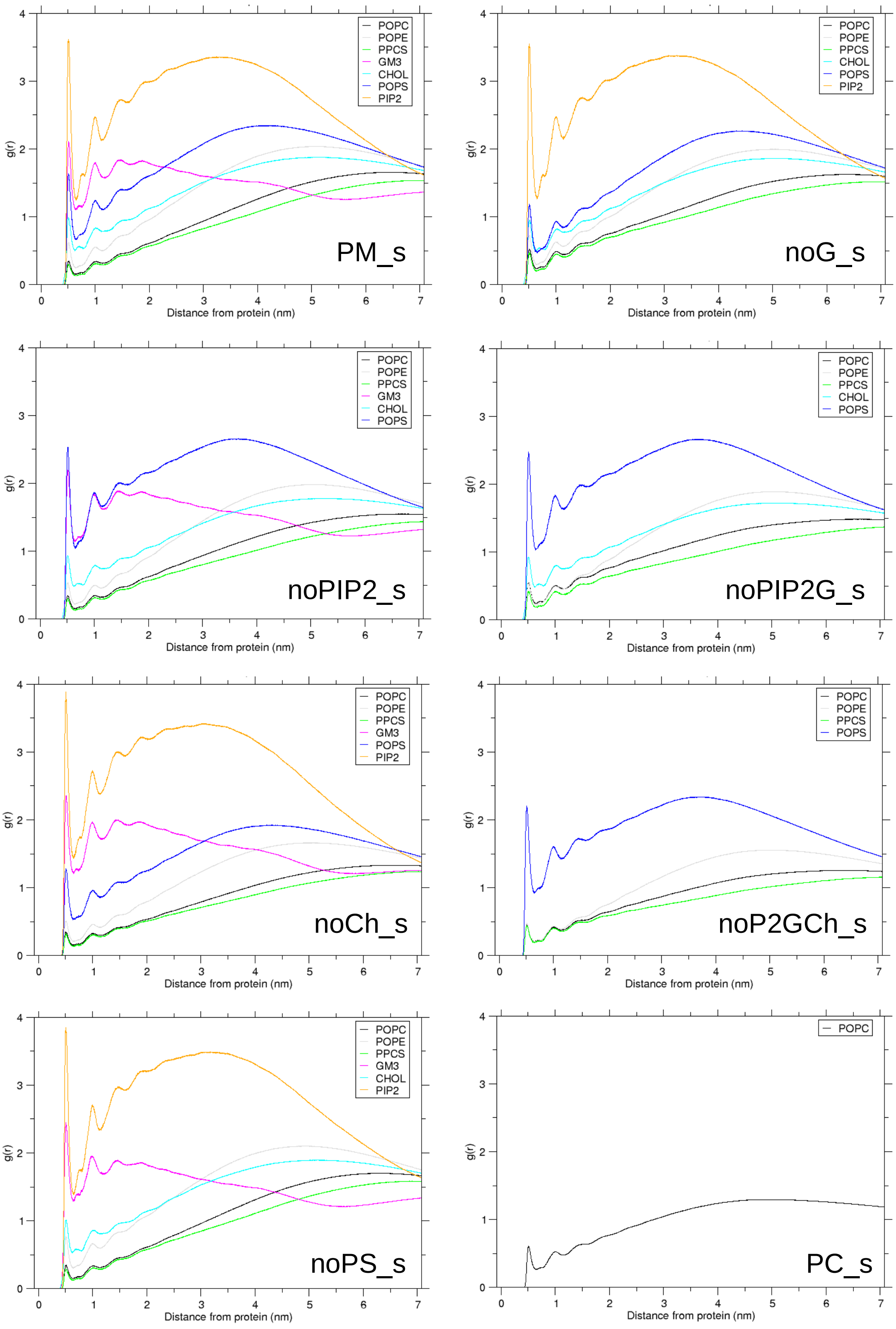

Figure S2. Radial distribution of lipid species about Kir2.2 in all systems simulated containing 9 Kir2.2 channels (see Table S1), taken from the first 20 μs of simulation. Radial distributions in each system are shown for each lipid in the system: PIP_2 is shown in orange; POPS in blue; GM3, magenta; Cholesterol, cyan; PPCS, green; POPE, pale grey; POPC, black.

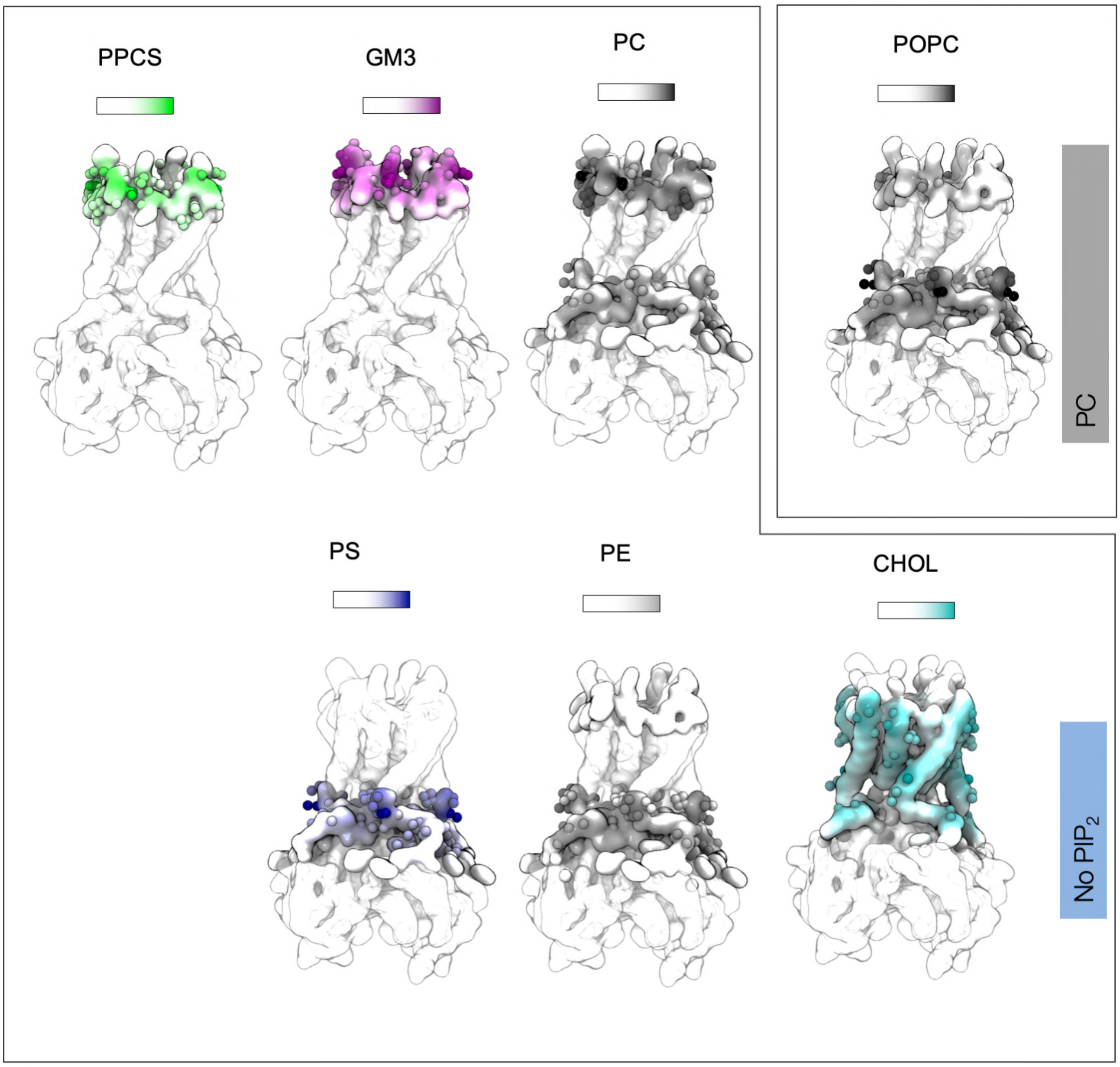

Figure S3. The frequency of lipid headgroup interactions at each protein residue are shown on the structure of the Kir2.2 channel for all lipids present in the *Large System No PIP*$_2$ and *PC* simulations (containing 144 Kir2.2 channels, see Table S1 for details). Interaction of lipid headgroups are mapped onto the protein surface with the frequency of interaction at each residue coloured on a sliding scale (transparent = no interaction; white = lowest interaction frequency; coloured = high interaction frequency). Residues that interact with a frequency > 2.5 % of the total lipid headgroup interactions (for a given lipid type) are show with side chains beads as spheres. The definition of lipid headgroups is detailed in the Methods section.

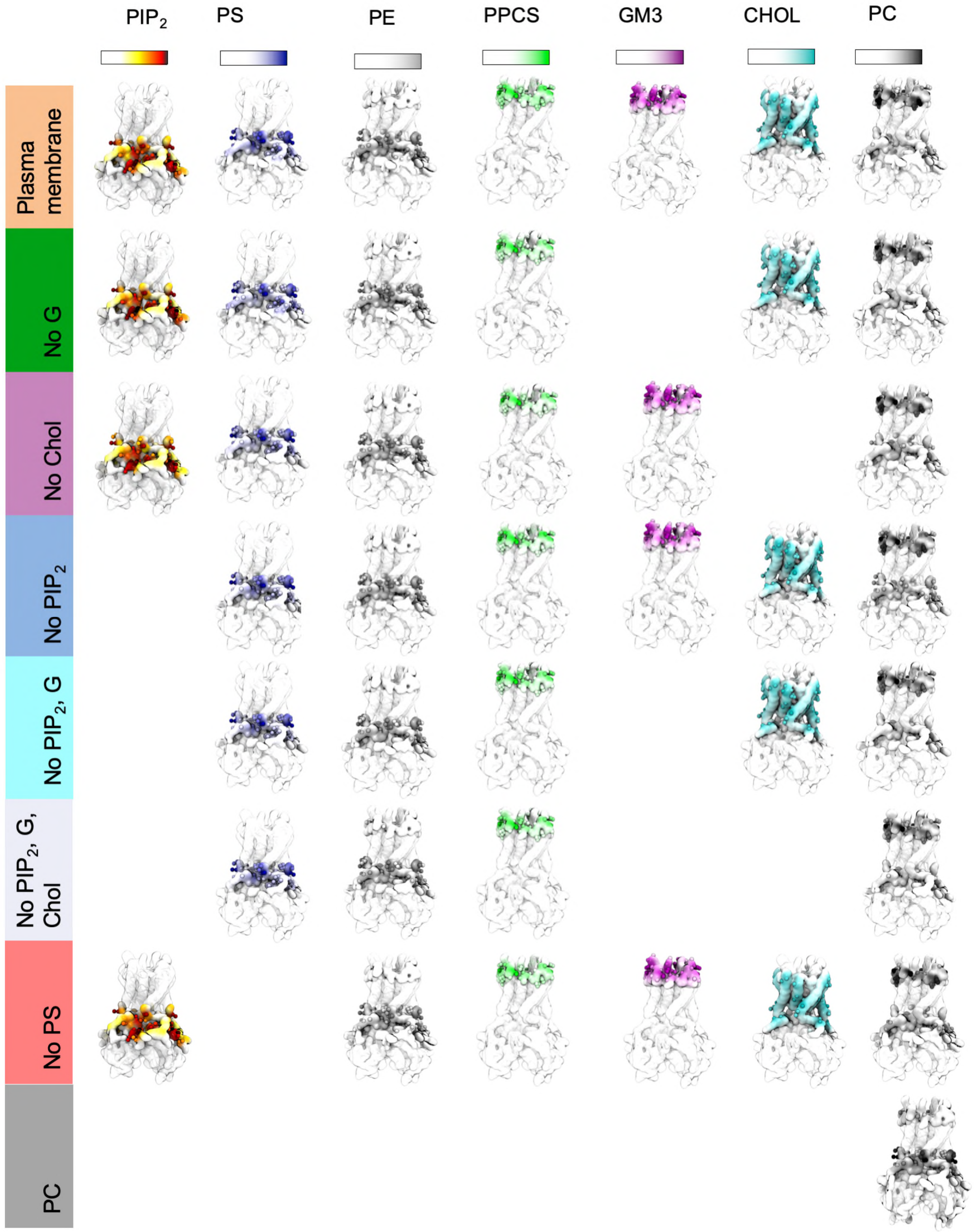

Figure S4. Frequency of lipid interactions at each protein residue for all lipids present in the simulations containing 9 Kir2.2 channels. Interaction of lipids headgroups are shown on the protein surface with the frequency of interaction at each residue coloured on a sliding scale (transparent = no interaction; white = lowest interaction frequency; coloured = high interaction frequency). Residues that interact with a frequency of > 2.5 % of the total lipid interactions (for a given lipid type) are show with side chains beads as spheres. See Table S1 for a details of the membrane lipid compositions.

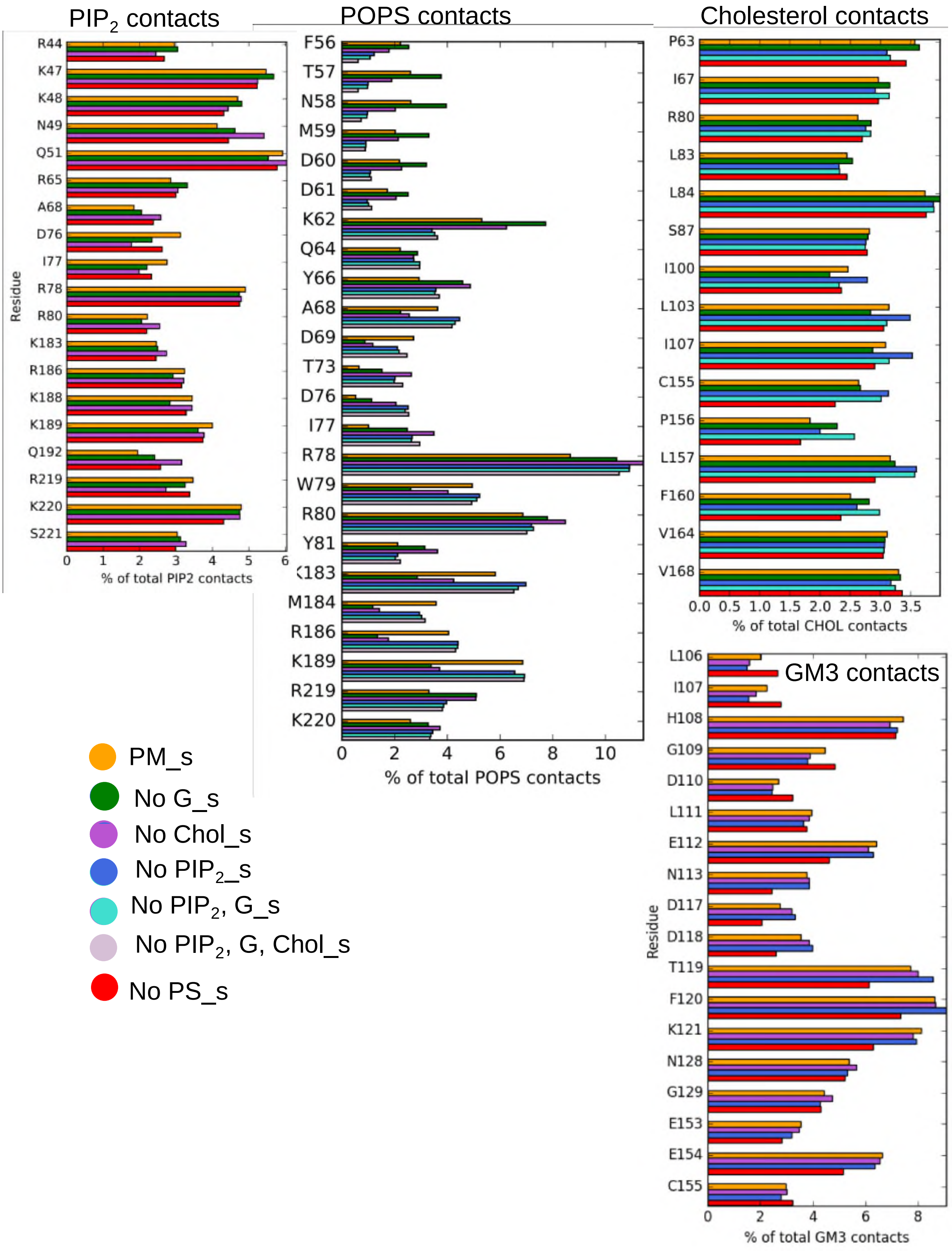

Figure S5. Comparison of interacting residues for PIP_2, POPS, cholesterol and GM3. Bar graphs show the interaction frequencies of those residues with > 2.5 % of the total PIP_2, POPS, cholesterol or GM3 interactions, in simulations of 9 Kir2.2. Simulations are coloured according to the key. More details of the membrane lipid compositions are shown in Table S1.

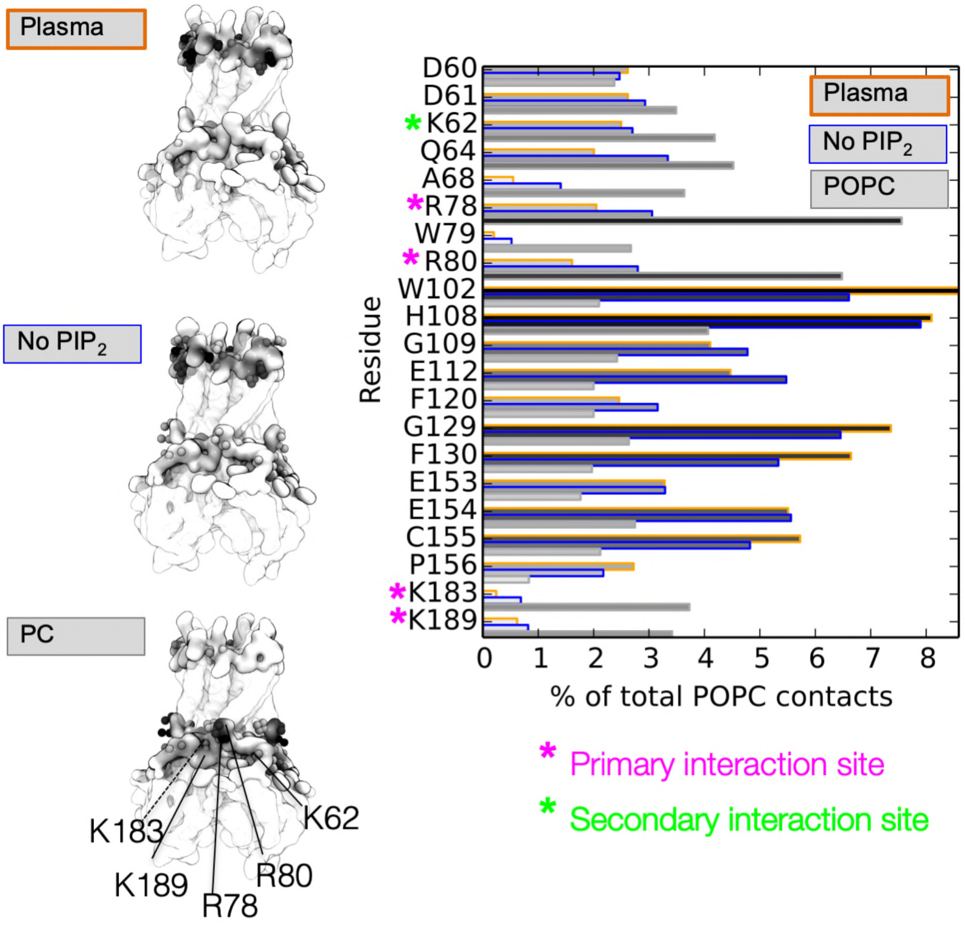

Figure S6. Comparison of PC – Kir2.2 interaction sites in the presence of PIP_2 and PS and other lipids ('Plasma'), in the presence of PS and other lipids ('No PIP_2') and in a membrane comprised entirely of PC ('PC') in simulations containing 144 Kir2.2 channels.

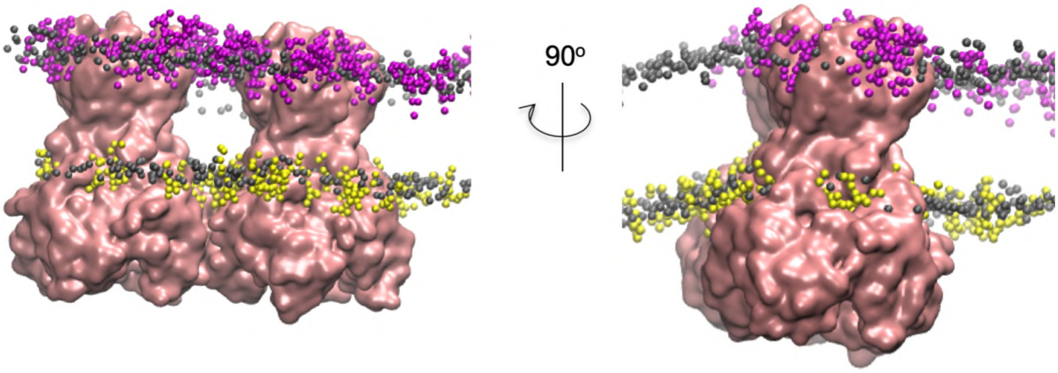

Figure S7. Snapshot of the position of phosphate groups of PC, PS and PE (grey), PIP_2 (yellow) and headgroup beads of GM3 (magenta) in the PM simulation. Two Kir2.2 channels are show as pink surfaces.

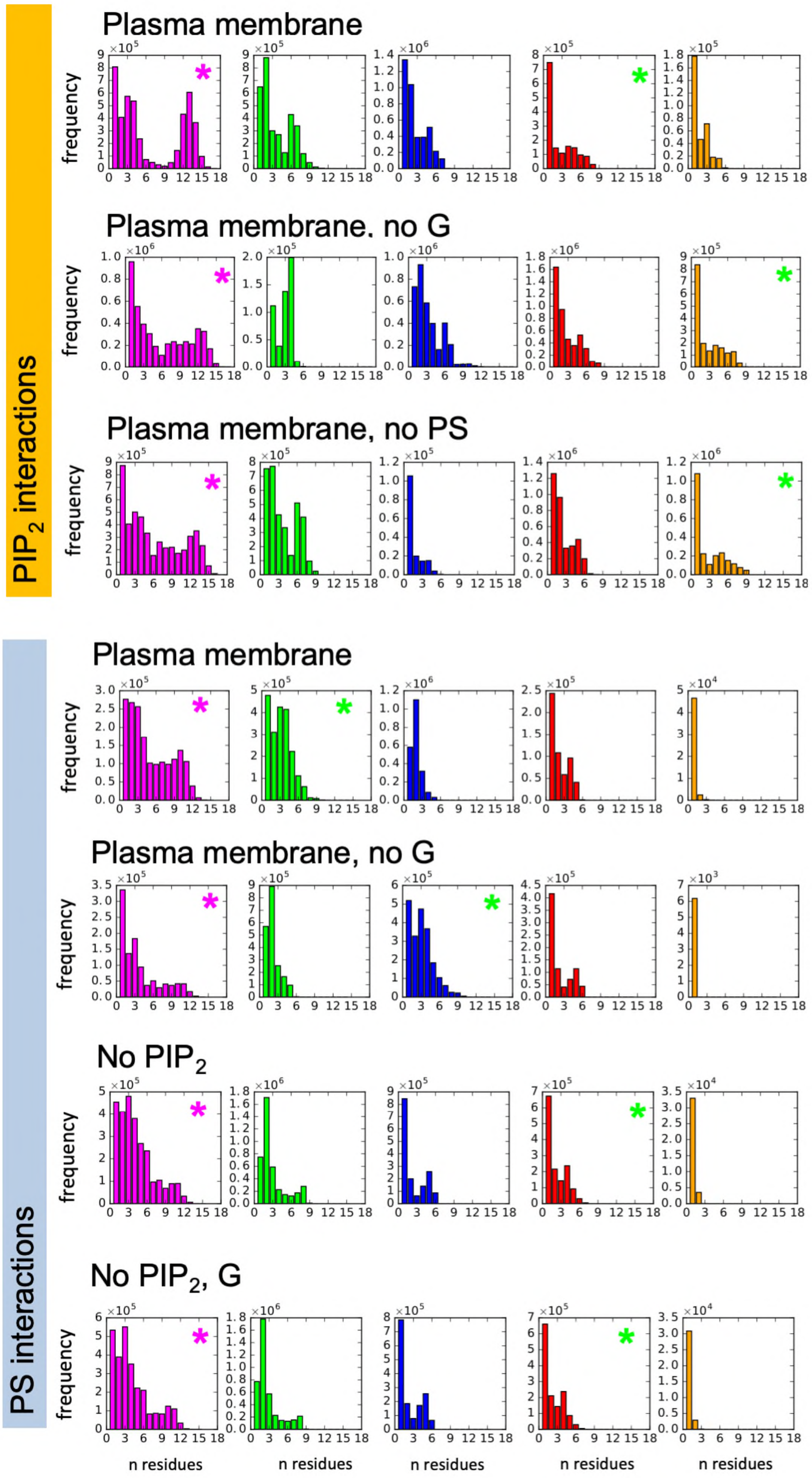

Figure S8. The number of contacts made simultaneously by a single lipid molecule when interacting at the interaction sites identified in Fig 4 (coloured as in Fig 4). The sites which coincide with the experimentally-determined primary and secondary interaction sites are indicated with a magenta or green asterisk, respectively.

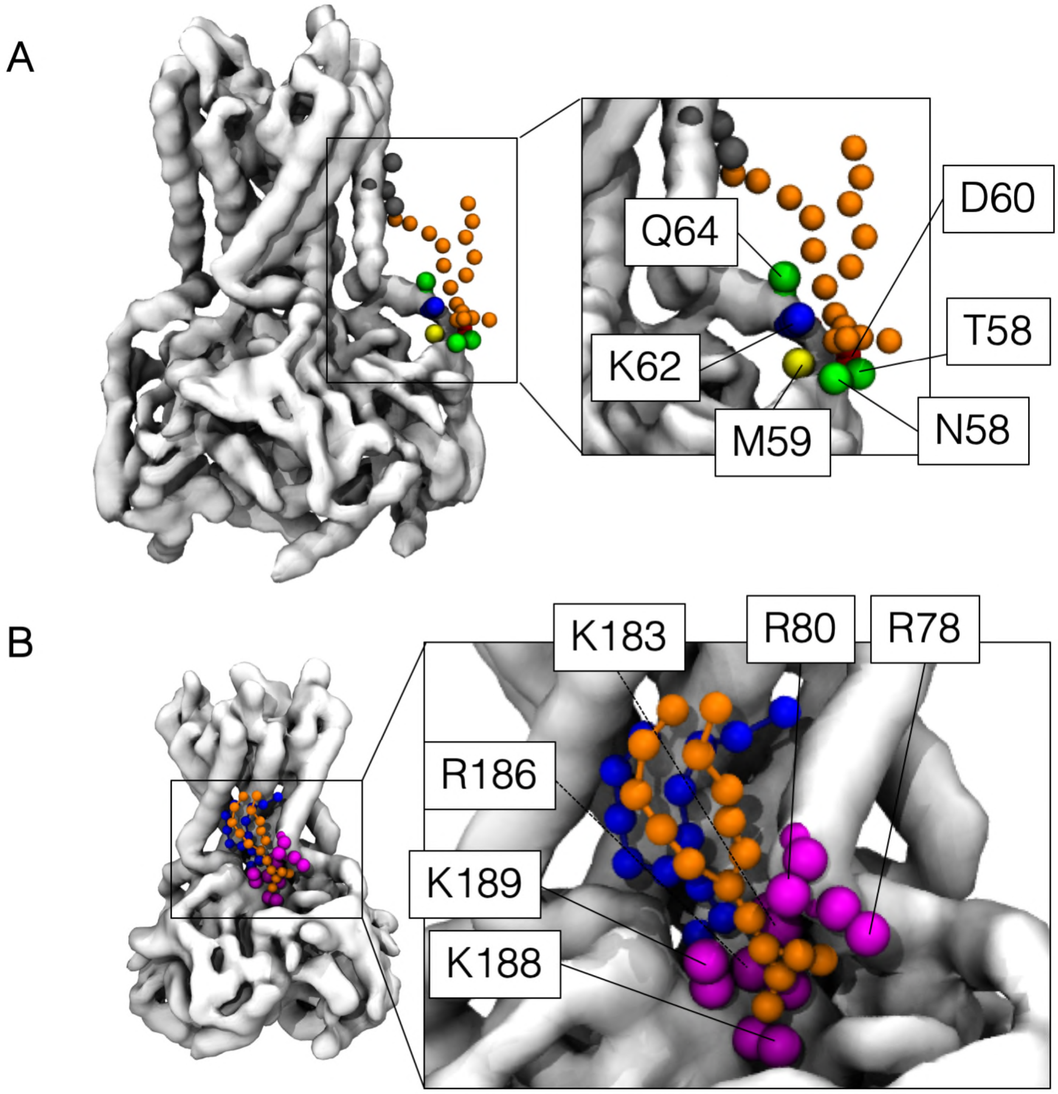

Figure S9. Snapshots of PIP_2 and PS molecules interacting at the primary and secondary interaction site of Kir2.2 in the Plasma membrane (*PM)* simulation. **A** Snapshot of PIP_2 (orange spheres) interacting at the secondary interaction site. An overview is on the left, and a zoomed view is on the right. Side chains of primary site residues are shown as pink spheres. Sidechains of residues interacting with PIP_2 are shown as spheres and coloured according to their chemical properties: polar (green), positively charged (blue), negatively charged (red) and methioinine (yellow). Not that only the backbone of D60 interacts with the $PIIP_2$ headgroup. **B.** PIP_2 and PS interacting at the primary interaction site, overview on the left, with zoomed view on the right. PIP_2 is shown as orange spheres and PS shown as blue spheres. Side chains of primary site residues that interact with the PIP_2 headgroup are shown as pink spheres.

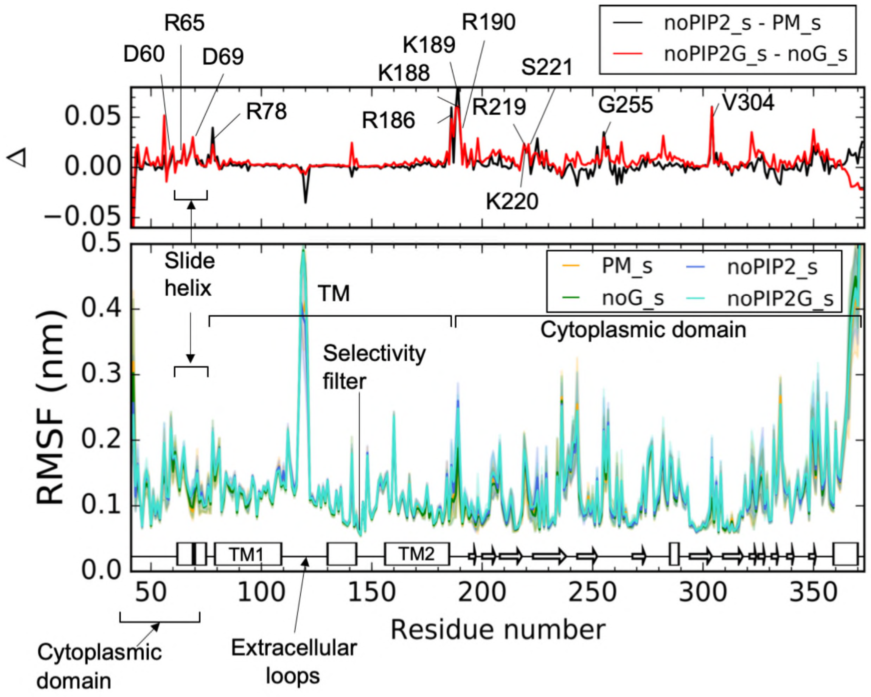

Figure S10. Flexibility of Kir2.2 channels in simulations containing PIP_2 (*PM_s*, *No G_s*) and those with no PIP_2 present (*No PIP_2_s*, *No PIP_2G_s*). The lower panel shows the root mean squared fluctuation (rmsf) of the entire protein, with features of the protein labelled, and secondary structure shown along the bottom of the figure (rectangle = helix; arrow = beta sheet). The rmsf is averaged over the course of each simulation, and averaged over each of the nine proteins in each simulations, and over all four monomers of each protein. The shaded regions around the line shows the standard deviation of this average. The upper panel shows the difference when PIP_2 is or is not included; the difference between the *No PIP_2_s* and *PM_s* simulations in black, and between the *No PIP_2G_s* and *NoG_s* in red. A positive difference indicates that the *No PIP_2_s* or *No PIP_2 G_s* channels are more flexible.
